# Multiphase OH Oxidation of Bisphenols: Chemical Transformation and Persistence in the Environment

**DOI:** 10.1021/acs.est.5c02030

**Published:** 2025-06-26

**Authors:** Jie Yu, Brandon Wu, Chao Peng, Jeremy Wentzell, Michael J. Wheeler, Joshua O. Osagu, Xianming Zhang, Li Li, Jonathan P. D. Abbatt, John Liggio

**Affiliations:** † Department of Chemistry, 7938 University of Toronto, Toronto, Ontario M5S 3H6, Canada; ‡ School of Public Health, 6851 University of Nevada, Reno, Reno, Nevada 89557, United States; § Air Quality Research Division, Environment and Climate Change Canada, Toronto, Ontario M3H 5T4, Canada; ∥ Department of Chemistry and Biochemistry, 5618 Concordia University, Montreal, Quebec H3G 1M8, Canada

**Keywords:** OH radical, BPA alternatives, multiphase oxidation, environmental persistence

## Abstract

Bisphenol A (BPA) is a common endocrine disruptor widely found in commercial products. Despite negative human health effects, its usage is not fully banned worldwide with ongoing human exposure from sources including dust, aerosol particles, and surfaces. Although attention has been paid to the abundance of alternatives with similar structures that are replacing BPA, uncertainties remain with respect to their chemical transformations and products, toxicity, and environmental fate. We provide the first experimental and modeling assessment of gas-particle multiphase OH oxidation of BPA and six common bisphenol alternatives. We examine the transformation of condensed-phase BPA and its alternatives using an oxidation flow reactor with products monitored by online mass spectrometry. Fourteen products were identified and used to develop a generic mechanism applicable to all bisphenols and to provide inputs into an environmental fate model (PROduction-to-Exposure; PROTEX). Our modeling results highlight the role of heterogeneous surface reactions in determining the indoor retention of these chemicals and their relative environmental persistence indoors and outdoors. All investigated parent molecules yield transformation products predicted to accumulate indoors, with extended indoor persistence if a long chemical lifetime on surfaces (e.g., >100 weeks) is assumed. Evidence of phenoxy radical presence upon oxidation raises a human health risk concern.

## Graphical abstract

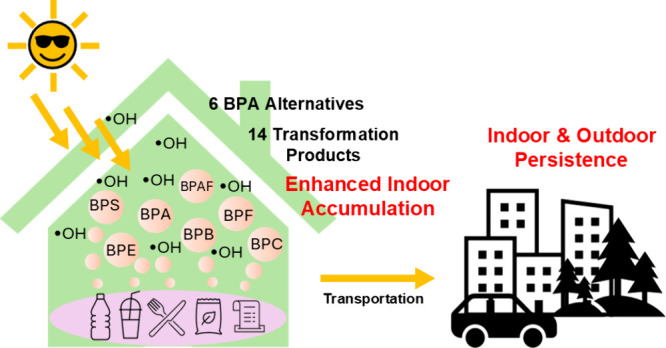

## Introduction

Bisphenol A (BPA) is an industrial chemical extensively used in the production of epoxy resins, polycarbonate plastics, and lacquer coatings.^1–4^ BPA is ubiquitous in consumer products such as food packaging, baby bottles and toys, thermal receipt paper, medical equipment, dental resins, etc.^5–8^ Its widespread prevalence in plastic products and estrogenic properties has long raised concern with respect to its threat to human health.^9–14^

BPA is one of the most potent endocrine-disrupting compounds (EDCs) which arises via strong binding to estrogen receptors.^15–18^ Numerous studies have implied impacts on hormone-related cancers, cardiovascular disease, obesity, infertility, insulin resistance and type 2 diabetes, and mellitus.^12,13,19–24^ BPA leaches from food packaging into food and beverages,^25^ and the manufacturing and consumption of BPA and associated products also leads to its ubiquitous presence in air, soil, water, and house dust.^25–29^ Over 95% of the world population is exposed to BPA, with prevalence in urine, blood, and breast milk.^19,30–34^ While BPA exposure via dietary ingestion accounts for 90% of the total exposure, predominantly from food packaging leachate and contamination,^35–38^ other exposure routes such as inhalation, dermal contact, and dust ingestion are also possible. As of December 2024, regulations on BPA have predominantly focused on its presence in the food industry, with banned BPA usage in baby contact products by regulatory agencies in North America and Europe.^36,39–43^

Industry is replacing BPA with new bisphenol chemicals in “BPA free” consumer products, namely, “BPA alternatives” such as bisphenol S (BPS), bisphenol AF (BPAF), bisphenol F (BPF), and bisphenols B (BPB). BPS, BPF, and BPAF are common, valued for their high heat and light resistance in plastics and coatings (BPS), increased durability in epoxy resins (BPF), and high reactivity as a cross-linker (BPAF).^44–47^ These alternatives are structurally similar to BPA, with replacement of the bridging hydrocarbon group and retention of the biphenolic backbone structure. With molecular similarities to BPA, some alternatives are found in most environmental compartments including the human body, and they exhibit similar or greater (for BPS and BPAF) endocrine-disrupting properties than BPA.^47–52^ Despite the increasing demand for BPA-free consumer products, the environmental fate, persistence, and toxicity of these alternatives remain unclear.

Bisphenols have short gas phase half-lives due to OH oxidation.^38^ However, longer overall lifetimes are expected due to their semivolatile nature and partitioning to airborne particles and other condensed-phase materials.^38,53,54^ Bisphenol-containing particles can be directly released into the air, predominantly from indoor origins, during the processing, production, and wear of consumer products or can arise from gas-particle phase partitioning. Bisphenols accumulate in indoor dust and organic surface films through particle settling or partitioning and may undergo oxidative transformations via multiphase reactions. The importance of multiphase chemistry occurring indoors has been recently recognized for its role in controlling the composition of indoor surface reservoirs, air, and aerosol particles.^55–60^ The partitioning and reaction time scales on indoor surfaces are significantly different than those outdoors.^55,58^ The physiochemical properties of the chemicals, environmental parameters, and characteristics of the surfaces are important factors in controlling indoor surface chemistry. Current studies have only focused on a few environment pollutants; BPA alternatives are yet to be explored.^55,56,58^

Well recognized outdoors, the OH radical also arises indoors from the ozonolysis of alkenes and photolysis of HONO.^61–65^ Indoor OH concentrations are typically on the order of 10^5^ molecules cm^–3^, but concentrations up to 10^7^ molecules cm^–3^ have been reported.^61,62,66–70^ Aqueous OH reactions of bisphenols have been examined for BPA and some alternatives in the context of wastewater advanced oxidation processes (AOPs).^71–74^ The only heterogeneous OH oxidation experiments were performed for BPA in liquid-semisolid sea-spray aerosol mimics.^75^ Biological impact studies on OH exposure to bisphenols are available only for BPA, with little characterization of product identity. This AOP oxidation mixture showed an enhancement in toxicity,^76–78^ metabolism inhibition,^78^ and estrogenic activity reduction,^77,79^ as compared to BPA.

This work addresses gas-phase OH oxidation of condensed phase BPA and six common alternatives, with a focus on identifying transformation products and proposing evolution pathways. This is motivated by the fact that regulations have mostly been based on environmental and health impacts of parent species, thus neglecting contributions from transformation products. The reactions were performed in an oxidation flow reactor (OFR), where the bisphenols were embedded in aerosol particles with environmentally relevant OH exposures. Online nontargeted analysis by extractive electrospray ionization time-of-flight mass spectrometry (EESI-TOFMS) was employed for product identification. A multimedia contaminant fate model “PROduction-to-EXposure” (PROTEX) evaluated the environmental persistence of the parent and transformation products indoors and outdoors,^80^ under the assumptions of constant indoor release and multiple removal pathways including surface reactions. The model also determines the primary reservoir in which each compound would reside as a function of a chemical’s lifetime on indoor surface compartments. Together, the results can guide subsequent toxicity and exposure analyses and any risk management action(s) if needed.

## Materials and Methods

### Multiphase OH Oxidation in an OFR

The reactions of gas-phase OH radicals and particle-phase bisphenols were studied individually for BPA, BPS, BPF, BPB, BPC, BPE, and BPAF (Table S1) in an OFR, with the experimental setup adapted from Liu et al.^81,82^ Ammonium sulfate ((NH_4_)_2_SO_4_, AS) particles were generated via atomization of a 5.3 mM aqueous solution and dried by being passed through a diffusion drier (model 3062; TSI). Once a stable AS particle concentration of 5–10 ug/m^3^ had been reached, pure bisphenols were heated in a temperature-controlled Pyrex tube. Bisphenol-coated particles were created when AS seed particles passed through the headspace of the heating tube. The heating temperature for each pure bisphenol was set to achieve a steady-state concentration of 20–45 ug/m^3^ for coated particles and a mode mobility diameter of 100–130 nm. The concentration of pure bisphenols ranged between 15 and 35 ug/m^3^ to reach an average coating thickness of approximately 8–20 nm. The particle size and concentration remained stable throughout the experiments (±5%). A list of heating temperatures and literature melting points for bisphenols is given in Table S1. A honeycomb-shaped activated carbon denuder (Aerodyne, Inc.) was attached downstream to remove volatile organic gases from the flow while transmitting bisphenol-coated particles. Particles were then sent through a 16 L cylindrical quartz oxidation flow reactor (OFR) along with an 8 L/min zero air flow at 45% relative humidity (RH), resulting in a particle residence time of approximately 2 min. Due to the low water solubility of bisphenols, the low RH after the drier (<10%, which is below the efflorescence RH of AS) and the 45% RH in the OFR (which is lower than the deliquescence RH of AS), the particles are expected to remain in a solid state throughout the reaction.^83,84^ A total of eight 254 nm UV lamps (Jelight Co., Inc.) were mounted around the OFR for adjustable UV irradiation intensity. The lamps were covered by a quartz sheath tube and purged with a total of 30 L/min air for heat dissipation. OH radicals were generated by the photolysis (2 UV lamps) of 2 ppm of O_3_ under the presence of water vapor. At steady state, an OH exposure of 3.3 × 10^11^ molecules s cm^–3^ was achieved in the OFR, as determined by measuring the loss of CO (Text S4). At the exit of the OFR, parameters including the particle size distribution, RH, ozone concentration, and organic concentration were measured by a scanning mobility particle sizer (model 3936; TSI), a RH sensor (model HMP 60; Vaisala Inc.), an O_3_ analyzer (model 202; 2B Technologies) and a high-resolution time-of-flight aerosol mass spectrometer (HR-TOF-AMS; Aerodyne Inc.), respectively. A schematic diagram of the OFR system is provided in Figure S1.

### Online Monitoring by EESI-TOFMS and Identification of Transformation products

The formation of transformation products was monitored online by an extractive electrospray ionization time-of-flight mass spectrometer (EESI-TOFMS; Aerodyne, Inc.), which was connected to the OFR exit flow. The instrument inlet is coupled closely to the exit of the OFR. The chemicals are expected to remain intact during the transfer period (<1 s). EESI-TOFMS uses a soft ionization technique to provide the chemical composition of particulate organic compounds in real time.^85^ The EESI inlet was sampled at 300 cm^3^/min through a honeycomb-shaped activated carbon denuder (Aerodyne, Inc.) for volatile gas removal. The electrospray solution was a water–acetonitrile (ACN) (20:80 by volume) solution, doped with 200 ppm of NaI as the charge carrier to promote ionization by sodium ion (Na^+^) adduct formation. The solution was transported by a nano electrospray emitter (Fossil Ion Technology; 50 μm ID, 50 cm length) to promote collision of droplets with organic particles in the flow.^85^ The EESI-TOFMS was operated in positive ion mode with a potential difference of +2.55 kV between the EESI probe and the mass spectrometer (APi-TOFMS). Analytes were largely observed as sodium adducts [M]·Na^+^, whereas ACN from the working solution gave rise to a minor adduct [M­(ACN)]·Na^+^ for some species, with an abundance of 8–15% of the parent ion adduct [M]·Na^+^. Cluster ions with the formula [(NaI)_*x*_(H_2_O)_*y*_(CH_3_CN)_*z*_]•Na^+^ were used for mass calibration across the *m*/*z* range of 20–400. Various factors such as particle size, particle solubility and the choice of the electrospray solution influence the ion sensitivity in the EESI-TOFMS system.^85^ The oxygenated products are expected to be more soluble in the electrospray solution due to the increased polarity, thus, enhancing their detection. Supplementary offline filter sampling and high-performance liquid chromatography-electrospray ionization-high-resolution mass spectrometry (HPLC-ESI-MS) analyses were also performed for the oxidation mixture of BPA and BPS. Details are provided in the SI.

### Environmental Transport and Persistence prediction by PROTEX model

We evaluated the environmental fate and transport of bisphenols and their transformation products using the PROTEX model,^80,86,87^ with the same environmental parametrization as in Miramontes and Li (2023).^80^ PROTEX simulates the distribution of bisphenols and transformation products across multiple indoor compartments, the loss via reactive surface reactions and human cleaning activities, as well as transport between the indoor, urban and rural scales of a region.^86^ Bisphenols and transformation products are assumed to be constantly released into indoor air at a unit emission rate (e.g., 1000 kg/year). Once released, these chemicals undergo indoor partitioning and removal processes, and they are subsequently ventilated into the urban environment and eventually reach the rural environment. Text S3 details the partitioning and reactivity properties of the modeled chemicals used in PROTEX modeling.

Past studies have shown heterogeneous surface reactions are important indoors.^88–91^ However, the rates of heterogeneous surface reactions for bisphenols and their transformation products have not yet been experimentally determined or computationally predicted. Therefore, we adopted three assumed chemical lifetimes on surface compartments (1, 10, and 100 weeks), defined as the average residence time of a chemical on artificial surface compartments indoors (carpet, vinyl flooring, hard surfaces, and walls and ceilings) and outdoors (urban impervious surfaces) to explore the sensitivity of modeled results to uncertainty in heterogeneous surface reactions. Notably, we assumed that bisphenols and all their transformation products share the same chemical lifetime on surface compartments.

We computed two indicators to evaluate the multimedia mass distribution and environmental persistence of assessed chemicals:^80^

(A) Indoor chemical mass distribution (C_m_,%) is defined as the percentage of steady-state chemical mass found in all indoor compartments (M_indoor,1_ + M_indoor,2_ + . . . + M_indoor, n_, equivalent to $\overset{\mathrm{indoor},n}{\underset{i=1}{\sum }}M_{i}$ ) across the defined totality of the regional environment (eq 1):
1 $$C_{m}=\frac{\overset{\mathrm{indoor},n}{\underset{i=1}{\sum }}M_{i}}{\overset{\mathrm{indoor},n}{\underset{i=1}{\sum }}M_{i}+\overset{\mathrm{urban},n}{\underset{i=1}{\sum }}M_{i}+\overset{\mathrm{rural},n}{\underset{i=1}{\sum }}M_{i}}\times 100$$
(B) Overall persistence (P_ov_, hours) indicates the overall lifetime in the indoor (P_ov,i_) or outdoor (P_ov,o_, the rural environment is illustrated here as an example) environment. It is calculated by dividing the total steady state mass across all related compartments by the total steady-state fluxes (F_1_ + F_2_ + . . . + F_m_, equivalent to $\overset{m}{\underset{i=1}{\sum }}F_{i}$ ) of loss processes and the outgoing advection from the environment of interest (eqs 2 and 3):
2 $$P_{\mathrm{ov},i}=\frac{\overset{\mathrm{indoor},n}{\underset{i=1}{\sum }}M_{i}}{\overset{\mathrm{indoor},m}{\underset{i=1}{\sum }}F_{i}}$$
3 $$P_{\mathrm{ov},o}=\frac{\overset{\mathrm{rural},n}{\underset{i=1}{\sum }}M_{i}}{\overset{\mathrm{rural},m}{\underset{i=1}{\sum }}F_{i}}$$

Additional details on model specifications and indicators are provided in Text S3.

## Results and Discussion

### Observation of Transformation Products

Each bisphenol was oxidized in the OFR individually, with an OH exposure level of 3.3 × 10^11^ molecules s cm^–3^ (see details in Text S4). Assuming the amount of exposed OH is linearly proportional to OH concentration and time, the equivalent exposure time outdoors is about 2–7 days (Text S5), with the global mean OH concentration in the range of 6 × 10^5^ to 1.6 × 10^6^ molecules cm^–3^.^92–94^ Indoor OH concentration may vary significantly depending on human activities, the presence of chemicals, and light levels. Current indoor models and measurements provide a typical indoor OH concentration range of 1.7–4.0 × 10^5^ molecules cm^–3^ when no major perturbations have occurred in the space.^62,69,95–98^ With our current OH exposure level in the OFR, the equivalent exposure time indoors varies from 10 to 22 days. This is a representative exposure time frame on indoor surfaces and can be much longer in many indoor conditions.

Identification of the transformation products was conducted by observing changes in ion signal time series upon the formation of OH in the OFR. A distinct decay of the parent ion signal and a rise of multiple product ion signals for most species was captured by EESI-TOFMS in real time. Figure 1 presents a sample time series of bisphenol S (BPS) and multiple OH transformation products, where each transformation product is labeled as the net gain or loss of hydrogen and oxygen atoms from the parent compound BPS, e.g., +1O, +1O-2H, etc. Significant parent compound consumption or generation of products was not observed for two control experiments, where only 2 ppm of O_3_ (O_3_ only period) or two UVC lamps (UV only period) were used in the OFR. The molecular formula of each major transformation product was identified by EESI-TOFMS.

**Figure 1:**
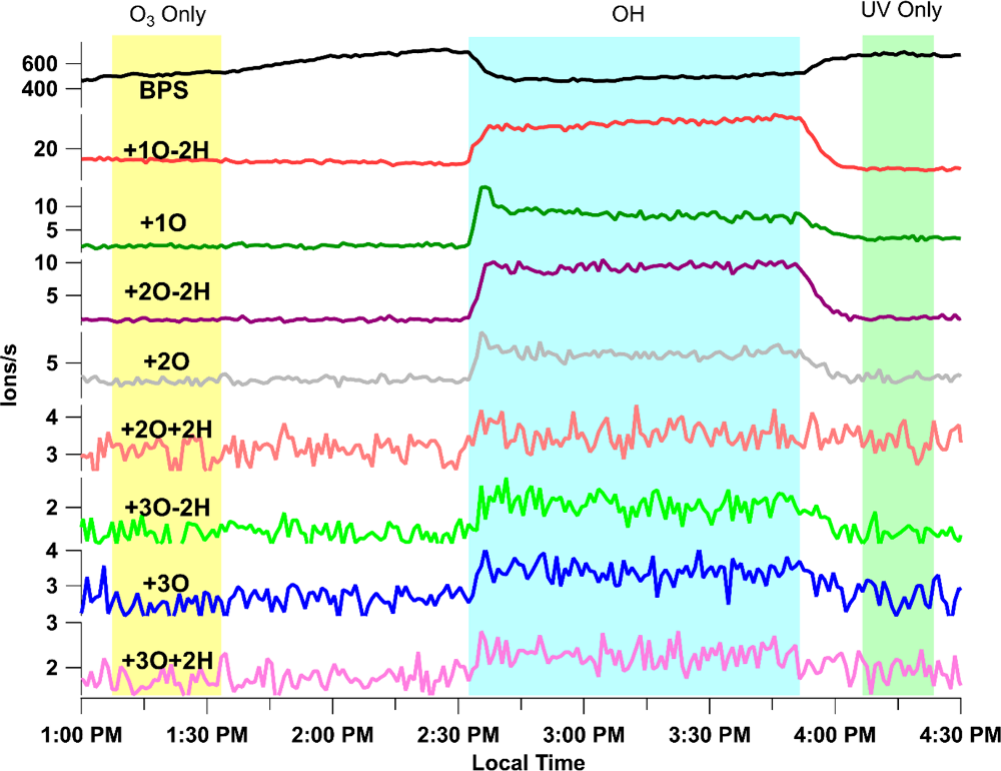
Time series of BPS and the observed transformation products during the control (O_3_ only and UV only) and OH exposure periods. Decay and rise of ion signals were observed for BPS and products, respectively, only during OH exposure. The signal increase of parent BPS before OH exposure is due to the slight temperature variation of the heating tube for BPS. The OH exposure was not applied until the set temperature had been reached again and the BPS signal remained stable.

The proposed identities are provided in the section below. Due to the variation of solubility in the electrospray working solution and the amount of product generated, the transformation products observed by EESI-TOFMS varied among bisphenols; i.e., the distribution of products detected for one bisphenol may not be the same as for other bisphenols. Regardless, important generalizations for transformation products of bisphenols or structurally similar compounds can be drawn based on the seven bisphenols examined. Specifically, when one oxygen atom from OH oxidation was added to the parent molecule, mass spectral features at +1O (i.e., addition of one oxygen atom to the parent molecule) and +1O-2H were observed; when two oxygen atoms were added to the parent molecule, multiple products including +2O-4H, +2O-2H, +2O and +2O+2H were observed. A similar product series was observed when three oxygen atoms were added to the parent molecule. In addition, +4O, +4O+2H, +5O, and +5O+2H were high molecular-weight products observed for some bisphenols (BPA, BPF, BPB, BPC, and BPAF; see Table S2). Note that structural isomers cannot be differentiated based on the EESI-TOFMS analysis. The transformation products predominantly remain in the particle phase due to increased intermolecular interactions via their higher oxygenation and polarity and the consistent particle size distribution measurements before and after oxidation. We acknowledge the possibility that further multigeneration oxidation steps may lead to fragmentation, producing more volatile, lower molecular weight compounds that could partition into the gas phase.^99^ Furthermore, the AS solution in the atomizer was slightly acidic (pH 5.8), and the particles are expected to be more acidic upon atomization and drying. This ensures that the bisphenol precursors (p*K*_a_ in the range of 8–10) exist in the neutral, nondissociated state. The chance of different products forming if the experiments had been done at a higher aerosol pH with dissociated reactants cannot be ruled out. A summary of the observed products for each bisphenol is provided in Table S2a, and the most probable structural isomer for each product is provided in Table S2b.

The BPA and BPS oxidation mixture and control samples (UV only and O_3_ only) were also collected and analyzed offline by HPLC-ESI-MS (Text S2). This work confirmed that some products only arise with OH oxidation, but the results were complicated by the offline nature of the analysis procedure.

Interestingly, we also saw evidence of phenoxy radical products, with a loss of one or three hydrogen atoms in the molecular formula (+1O-1H and +1O-3H, etc.). The proposed structures are provided in Table S3a. Phenoxy radicals are especially stable and persistent in condensed phases such as particulate matter and can act as reactive intermediates in reactions involving phenolic compounds, and are commonly classified as environmentally persistent free radicals (EPFRs).^100–105^ OH-initiated production of phenoxy radicals has been reported in aqueous phase measurements and gas-phase modeling work.^106–108^ In addition, phenoxy radicals have shown resistance to decomposition when oxidants are present and at high temperatures, primarily due to the stability of resonance structures that delocalize electrons through the aromatic ring and the phenolic oxygen.^109,110^ Given this information and considering the relatively short transit times (i.e., less than a second) from the OFR to the EESI inlet, phenoxy radicals are likely also produced in this multiphase OH oxidation process. While the mass spectral results are consistent with phenoxy radical formation, definitive additional measurements are required to confirm the identification. Lastly, a +CO product was also observed in the OH oxidation of multiple bisphenols (not observed in O_3_ only control; proposed structures provided in Table S3a). Our previous study on ozonolysis of BPA also found a +CO product and confirmed its identity as an aldehyde, with a formyl grouping added on the aromatic ring.^91^ Although the +CO product here is expected to also be an aldehyde, its formation pathway is unclear.

Another way to illustrate the evolution of bisphenols upon OH exposure is via the difference between EESI-TOFMS spectra collected for oxidized and unoxidized bisphenol particles. The differential mass spectrum of BPS, obtained by taking the difference in average ion signal intensity between the OH exposure period and the period immediately before OH exposure, is shown in Figure 2. The major reagent ion and adduct signals (Na^+^ at *m*/*z* 23; ACN·Na^+^ at *m*/*z* 64) remained stable throughout the monitoring period. A list of proposed structures of observed transformation products is provided in Table S2b. For BPS, the substantial decrease in parent ion signals at *m*/*z* 273 ([M]·Na^+^) and 314 ([M­(ACN)]·Na^+^) aligns with the BPS degradation observed upon OH exposure in the time series (Figure 1). The transformation products identified in Figure 1 all demonstrated net increases in the signal intensity. Lower molecular weight products +1O-2H (*m*/*z* 287), +1O (*m*/*z* 289), and +2O-2H (*m*/*z* 303) exhibited the greatest signal enhancement upon OH exposure relative to the other products. This indicates that lower molecular weight products are likely the earlier-generation products and hence more prominent than those with higher molecular weight at one OH exposure. If the OH exposure in OFR were to increase, greater signal enhancement for higher molecular weight compounds might occur but the signal change may also be affected by the compound solubility in the working solution and the associated ion sensitivity. A differential spectrum for another bisphenol (Bisphenol AF, BPAF) is provided in Figure S2.

**Figure 2:**
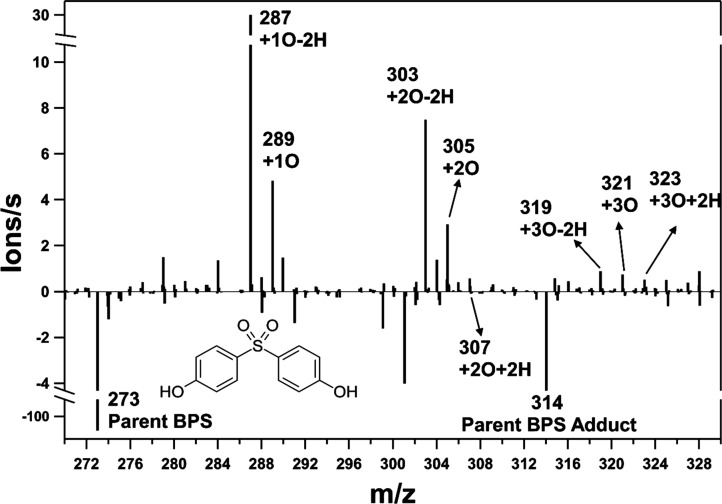
Differential positive ion EESI-TOFMS spectrum for BPS at OH exposure of 3.3 × 10^11^ molecules s cm^–3^. Based on the formula assignment, the main product peaks are labeled with the *m*/*z* ratio and gain or loss of C and O atoms with respect to the parent compound.

### Transformation Pathways

Guided by the literature on aqueous and gas-phase oxidation of aromatic and phenolic compounds, multiple transformation pathways are proposed for the multiphase OH oxidation of bisphenol compounds (Figure 3), with species that were detected by EESI-ToF-MS indicated by solid boxes. It should be noted that OH oxidation may also occur on other reactive sites on the aromatic ring or the bridging functional groups, depending on the type of bisphenols. The pathways proposed here are generalized for OH reaction with bisphenols (C_12_H_10_O_2_X; BPX); only a selection of possible structural isomers is included in solid boxes.

**Figure 3:**
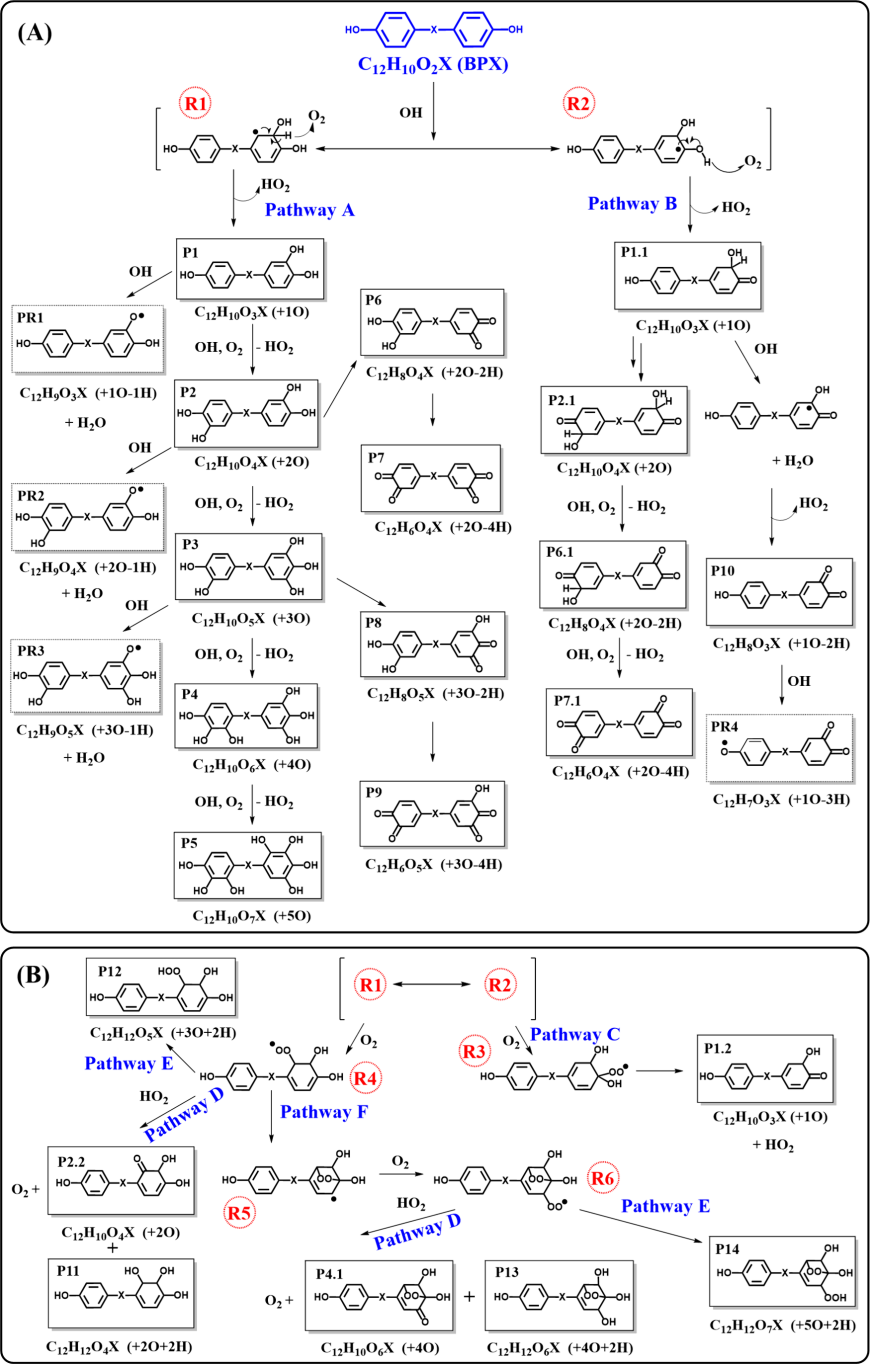
Proposed transformation pathways for bisphenols (BPX) upon OH oxidation, based on the observations for all the bisphenols assessed in this study. Followed by the production of intermediate resonance structures (R1 and R2) via OH addition, panel (A) involves pathways A and B that involve H atom abstraction and the formation of byproduct HO_2_ with the presence of O_2_, whereas panel (B) shows pathways C–F that are initiated by the formation of peroxy radicals (R3 and R4). Closed shell products observed by EESI-TOFMS are in solid boxes (P1–P14). Isomeric products are labeled as P X.X. Stable phenoxy radicals are in dashed boxes (PR1–PR4). Intermediate radical species are labeled as R1–R4. Note that structural isomers other than those indicated are possible. A summary of the proposed structures and associated pathways is provided in Tables S2b and S3a.

The first step of atmospheric degradation of phenolic compounds with OH radicals involves OH addition to the aromatic ring or H atom abstraction of the H atom on the phenol group.^111–117^ Gas-phase studies show that approximately 90% of the OH radical reactions preferentially proceed by addition to the aromatic ring.^111,116,118–122^ Thus, the major intermediate species formed via OH addition are hydroxy cyclohexadienyl radicals.^114–116^ Figure 3 shows an example of OH addition to the ortho position of one aromatic ring, providing two intermediate resonance hydroxy cyclohexadienyl radical structures (R1 and R2). From there, two pathways are proposed, as shown in Figure 3(A). Via Pathway A, starting with R1, an H atom may be abstracted by O_2_, to form the +1O product (P1). Further oxidations via the same pathway can generate +2O, +3O, +4O, and +5O products (P2–5, respectively) on the same or different aromatic rings of the bisphenol molecule. Further transformations on P2 and P3 can involve O_2_ converting *o*-dihydroxy molecules into *o*-quinones (+2O-2H and +3O-2H products; P6 and P8). Such conversion has been observed during the autoxidation of catechol to 1,2-benzoquinone,^123^ and the formation of quinones from OH oxidation of aromatic rings is commonly observed.^124,125^ Products +2O-4H (P7) and +3O-4H (P9) may be generated if the same oxidation occurs on the other aromatic ring. As discussed earlier, stable phenoxy radicals, formed via H atom abstraction from the hydroxyl group by OH radicals, can pass through the OFR and be detected by EESI-TOFMS. The presence of PR1–3 (in dashed boxes) was observed for some bisphenols used in this study (more details in Table S3). Note that as proposed by previous aqueous-phase oxidation studies,^126–128^ product isomers resulting from ring opening may also arise from multigeneration chemistry under aggressive oxidation conditions. Some examples of the ring-opening structures are provided in Table S5.

Similar to Pathway A, Pathway B consists of a hydroxyl H atom abstraction by O_2_ from the other resonance structure (R2), forming a +1O isomeric product (P1.1) with a carbonyl C=O group. A +2O product (P2.1) would be produced if the same mechanism occurs for the other aromatic ring. Subsequent H atom abstraction by OH radical (from the C–H bond) and O_2_ (from the O–H bond) may proceed, along with the elimination of H_2_O and HO_2_, respectively, to generate *o*-quinone products +1O-2H (P10), +2O-2H (P6.1), and +2O-4H (P7.1). From P10, an H-subtraction from the O–H bond may result in the phenoxy radical +1O-3H (PR4).

In additional to Pathways A and B, alternative pathways involving the generation of peroxy radicals can be proposed for both resonance structures R1 and R2, as shown in Figure 3B. Via Pathway C, O_2_ may also transform the hydroxy cyclohexadienyl radical (R2) to another +1O isomer (P1.2), via a peroxy radical (R3) at the ipso position and an HO_2_ elimination.^108,114^ Similarly, upon transformation of R1 into a peroxy radical (R4) by O_2_ (Pathway C), three additional pathways are possible. The peroxy radical (R4) can undergo radical self-reaction by the well-known Russell mechanism (Pathway D) to produce +2O (P2.2), +2O+2H (P11), and O_2_ or be converted to hydroxyl hydroperoxide (+3O+2H; P12) via pathway E.^129,130^ Lastly, via Pathway F, higher molecular weight products may be generated if R4 isomerizes to a carbon-centered radical (R5) and then reacts with O_2_ to transform into a bicyclic peroxy radical (R6), which has been identified as a key OH-initiated oxidation intermediate for multiple aromatic compounds.^131,132^ From R6, a set of products including bicyclic carbonyl (+4O; P4.1), bicyclic alcohol (+4O+2H; P13), and O_2_ can be obtained if two R6 radicals react via the Russell mechanism (Pathway D); +5O+2H product (P14) can be generated via Pathway E.

It is worth noting that the analytical technique employed in this study measured primarily products with higher molecular weights than the parent bisphenols, and thus, the proposed transformation pathways focus exclusively on these products. However, past studies on bisphenol removal in the aqueous phase have identified additional smaller products resulting from intense oxidation and fragmentation.^128,133^ For example, Schober et al. proposed that an OH attack on the bridging functional group (i.e., C–C or S–C bond) can initiate the formation of monophenolic complexes. While the production of these compounds in this system cannot be entirely ruled out, our EESI-TOFMS results do not provide strong evidence for their presence under the conditions of the current specific experiments.

Additionally, Cope et al. revealed that sulfate anion radical (SO4•−), generated under irradiation in the aqueous phase, can promote degradation of organic complexes even when OH is the intended radical source.^134^ While the ammonium sulfate seed particles were not hydrated in the current system (at 45% RH), this study nevertheless pointed out alternative oxidation pathways involving the initiation by sulfate radicals for organic particles when sulfate is present and in highly humid atmospheric conditions.

### PROTEX Model Results

PROTEX predicts that, among the seven investigated bisphenols, the transformation products of BPS and BPAF exhibit two strikingly different environmental behavior patterns, whereas the product behaviors of other bisphenols are similar to either those of BPS or BPAF. Therefore, BPS and BPAF, along with BPA-related products, are used as illustrative examples in this section; the results for other bisphenols (BPB, BPC, BPE and BPF) are provided in the SI. Although isomers (P X.X in Figure 3) were not modeled, minimal differences in modeled results would be expected, given their structural similarities. Phenoxy radicals (PR1–4 in Figure 3) were excluded from the evaluation, because their lifetimes remain unknown. Note that although the transformation and transport mechanisms of compounds in the indoor and outdoor environments vary, as reflected by the independent and uncoupled loss rates in the model, the identities of the oxidation products were assumed to be the same regardless of the form and location of the heterogeneous reactions.

Figure 4 shows that BPS and its transformation products possess the highest C_m_, the indicator for indoor mass distribution, among all of the investigated chemicals. Their high octanol-air partition coefficient (*K*_OA_) values (Table S6) indicate a higher tendency for retention in the indoor environment, especially the apolar or weakly polar phases of indoor surface compartments. Similarly, their low *K*_OW_ values (Table S6) imply strong partitioning into the aqueous phase of indoor compartments, such as the moisture sorbed to carpets and building materials.^80^ In contrast, BPAF and its transformation products exhibit the lowest C_m_ because of the lowest *K*_OA_ and highest *K*_OW_ values among the investigated chemicals (Table S6). In addition, since C_m_ reflects the relative distribution of chemicals indoors and outdoors, chemicals with high biodegradation half-lives (HL_biodegradation_; Table S6) in outdoor environmental compartments, such as +2O-4H and +3O-4H compounds, tend to accumulate in outdoor surface compartments and therefore exhibit a low C_m_ (Figure S3 and Table S6).

**Figure 4:**
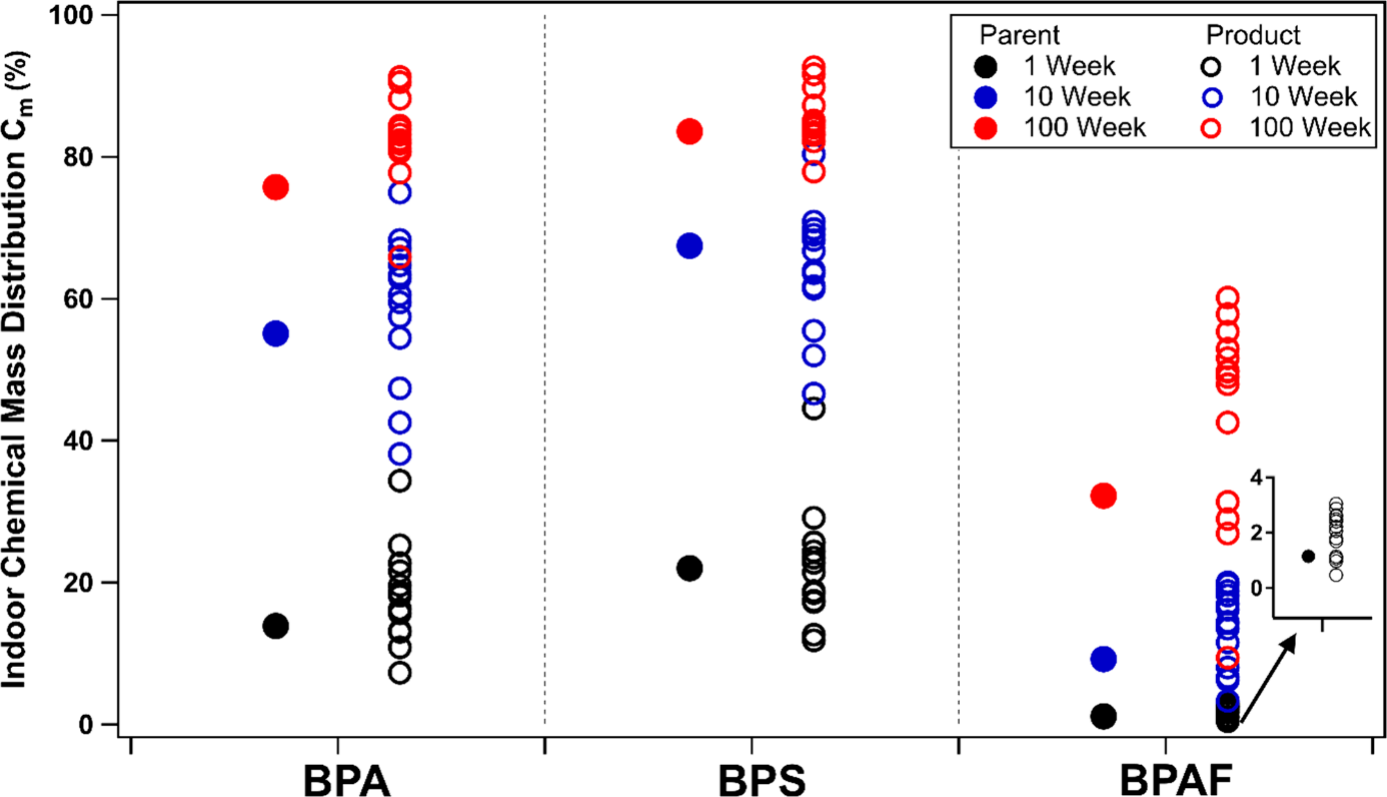
Indoor chemical mass distribution (C_m_,%), defined as the percentage of steady-state chemical mass found indoors in all compartments across the defined totality of the regional environment, for BPA, BPS, and BPAF-related closed shell compounds included in the mechanism in Figure 3. The C_m_ results obtained under the assumption of 1, 10, and 100-week surface lifetimes are shown in black, blue, and red circles, respectively. The C_m_ values for the parent bisphenols are presented in solid circles, whereas the products are presented in hollow circles. A compound-specific figure that differentiates each individual compound is provided in Figure S3. The C_m_ results for other bisphenols (BPB, C, E, and F) are provided in Figure S5.

Generally, more oxidized products tend to partition more readily into indoor surface reservoirs than do their parent compounds do. This is due to stronger surface interactions, as demonstrated by the predicted higher C_m_ values for many transformation products (hollow circles, Figure 4) relative to their parent compounds (solid circles of the same color). Our modeling further indicates that the indoor and outdoor fate of bisphenols and transformation products is highly sensitive to their assumed lifetimes on indoor surfaces. For example, when the surface lifetime is assumed to be 1 week, this rapid surface-bound reaction results in low C_m_ (<45% for all compounds), indicating that none of the compounds prefer to remain indoors. However, as the surface lifetime increases to 100 weeks, corresponding to slower surface-bound heterogeneous reactions, the chemical mass is more likely to accumulate indoors (C_m_ > 60% for most compounds). Exceptions are the BPAF-related compounds, which show consistently low C_m_ due to their high volatility, favoring outdoor distribution even at a 100 week surface lifetime. Overall, the critical role of lifetime on indoor surfaces highlights the importance of understanding and quantifying multiphase heterogeneous reaction rates of these chemicals.

In the multimedia environment, a chemical demonstrates higher overall persistence when a greater portion of its mass is distributed in a compartment where it has a long lifetime. Typically, chemicals degrade faster in the air compartment compared with condensed phase compartments. Thus, higher overall persistence (P_OV_) values can be found among chemicals with a greater proportion residing in the surface compartments indoors, and/or the soil and sediment compartments outdoors.^80,86^ Figure 5 shows the predicted overall persistence P_ov_ for indoor (P_ov,i_) and outdoor (P_ov,o_) environments for BPA, BPS, and BPAF and their transformation products. The magnitude of P_ov,i_ is strongly influenced by the assumed reaction lifetime on indoor surface compartments as heterogeneous surface reactions are the main mechanisms for the loss of these low-volatility compounds. In contrast, chemicals outdoors hardly accumulate on impervious outdoor surfaces with their primary residing compartments being soil and water. The rate of biodegradation (HL_biodegradation_) is the major factor influencing the magnitude of P_ov,o_, rather than the surface lifetime. This is reflected in the differences in P_ov,o_ for all surface lifetime scenarios, which are statistically insignificant, such that the P_ov,o_ of the same compound is not differentiable for various surface lifetimes (Table S6). When the surface lifetime is assumed to be 1 or 10 weeks, all investigated bisphenols and transformation products are predicted to be more persistent outdoors than indoors (higher P_ov,o_ than P_ov,i_). However, when the surface lifetime is assumed to be 100 weeks, most compounds are predicted to be more persistent indoors, with the exception of BPAF-related compounds. The investigated chemicals mainly reside in condensed compartments other than indoor and outdoor air. Thus, the P_ov_ results highlight that relative indoor and outdoor persistence is largely determined by the relative magnitudes of heterogeneous surface reactions and biodegradation, respectively.

**Figure 5:**
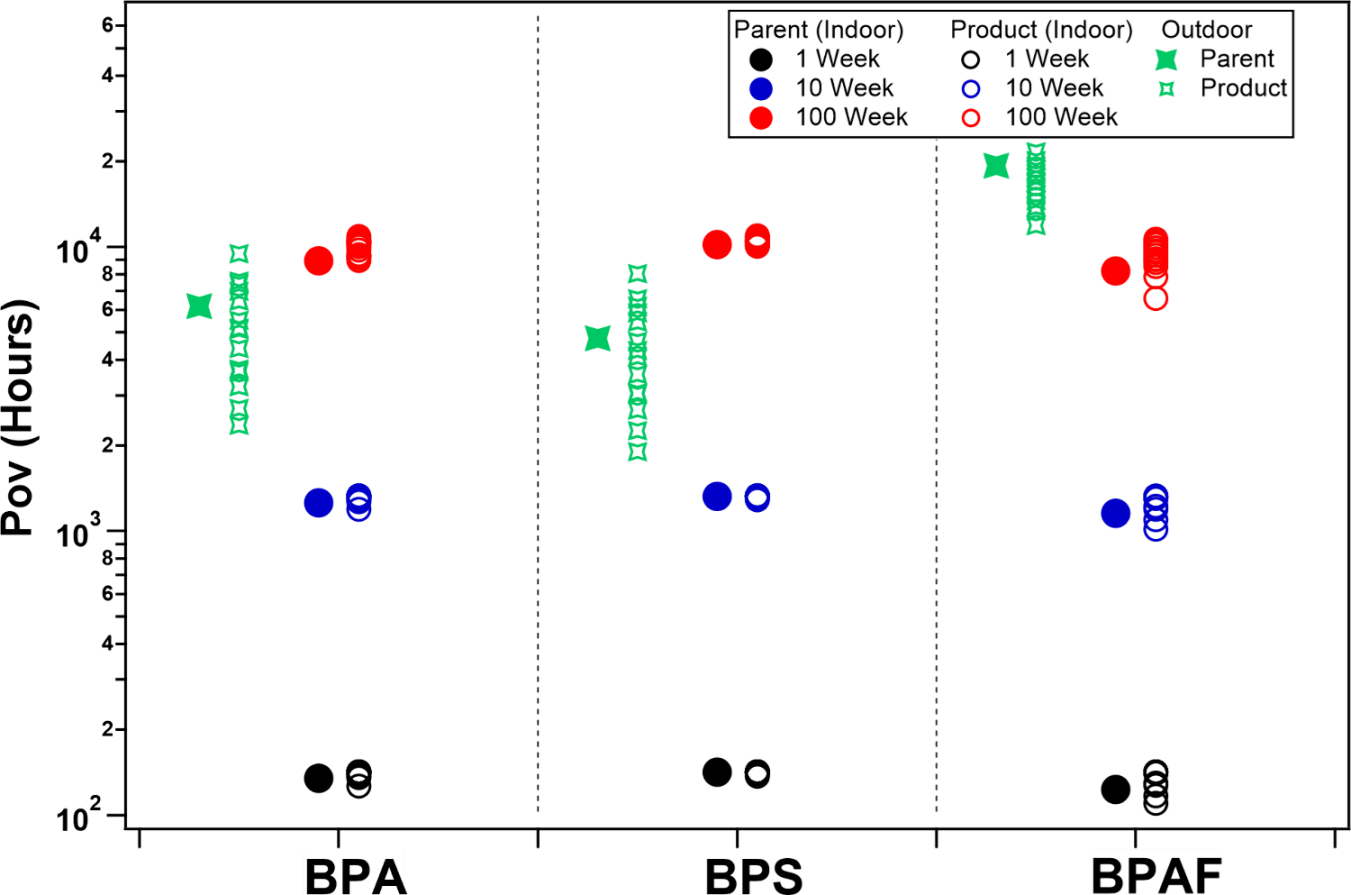
Indoor and outdoor (rural) overall persistence (P_ov,i_ and P_ov,o_, hours), defined as the overall lifetime in the corresponding environment, for BPA, BPS, and BPAF-related compounds. For persistence indoors, the P_ov,i_ results obtained under the assumption of 1, 10, and 100-week surface lifetimes are shown in black, blue, and red circles, respectively. The P_ov,i_ values for bisphenols are presented as solid circles, whereas the products are presented as hollow circles. For persistence outdoors, the P_ov,o_ values for bisphenols and products are presented as solid and hollow green stars, respectively. A compound-specific figure differentiating each individual compound is provided in Figure S4. The P_ov_ results for other bisphenols (BPB, C, E, and F) are provided in Figure S6.

Note that the model’s indoor environment was parametrized at 50% RH. Since relative humidity controls the aqueous phase volume in indoor surface compartments, an increase in relative humidity promotes the partitioning of hydrophilic, ionizable chemicals into surfaces such as the “walls and ceiling” (0.3% volume at 50% RH, increasing to ∼ 0.5% at 70% RH), thereby reducing their concentration in air and limiting their transfer to outdoor environments.^135^

### Environmental Implications

Despite organic contaminants being widespread in a variety of indoor and outdoor environmental compartments, our knowledge of human exposure to these contaminants remains limited. Moreover, with chemical transformations occurring in each environmental setting, the number and chemical complexity of organic contaminants are particularly large. While current studies are heavily focused on the abundance and partitioning behavior of a broad spectrum of commercial contaminants, the attention given to their transformation products and pathways and associated exposure and toxicity is comparatively limited. Knowledge on health and environmental impacts and transformation processes associated with transformation products is needed to support assessments and, if needed, actions to manage risks. Indoor environments are of particular interest given that many organic contaminants are emitted indoors. As well, they are especially complex due to low air-exchange rates, a wide variety of human activities, and the presence of multiple condensed phase reservoirs available for partitioning.^55^ Even though OH radicals are better known for promoting gas-phase reactions outdoors, there is the potential to react with OH radicals via gas-surface multiphase chemistry given that many contaminants are strongly partitioned to indoor surface reservoirs.^88,136,137^

This work is the first to investigate the multiphase OH oxidation of one of the most common indoor contaminant families, bisphenols. Whereas BPA has been regulated to some degree, six unregulated but widely used BPA alternatives were also studied. This work demonstrates that these molecules are prone to multiphase oxidation with OH radicals at environmentally relevant exposures, forming a complex suite of transformation products with the same backbone as BPA with potential health and environmental impacts.

The PROTEX multimedia model was applied to predict the environment-wide persistence of these transformation products. As compared with the properties of the parent compound, the transformation products are generally less volatile and more water-soluble, indicating enhanced partitioning into indoor organic surface reservoirs and hygroscopic compartments upon transformation. Model results revealed that all of the compounds studied have a strong tendency to remain indoors after emission and transformation, with a broad suite of the transformation products showing elevated indoor mass accumulation compared to the bisphenols. In the context of persistence, the relative magnitude of P_ov,i_ and P_ov,o_ is highly dependent on the relative magnitude of the assumed lifetime on indoor surface compartments and biodegradation in outdoor compartments. Lastly, this study demonstrates the presence of phenoxy radical products in the particles, which may potentially be EPFRs and contribute to respiratory and other health impacts.^104,105,138–140^

While this study has shown that OH oxidation can occur, recent work has shown that BPA can also react with O_3_ by analogous multiphase processes.^91^ These studies add to the growing body of evidence that many contaminants, including PAHs, phthalates, nicotine and THC, and a variety of oils, are all prone to multiphase oxidation processes under indoor conditions.^88,89,137,141–143^ The overall importance of indoor multiphase chemistry has been recently assessed by a modeling study, showing it to be likely important for molecules with log *K*_OA_ values larger than about 8.^144^ The log *K*_OA_ of our investigated chemicals lies above this threshold, thus highlighting the need for additional studies on the multiphase kinetics on different surfaces, the evolution of the early- and later-generation transformation products, and EPR analyses of oxidation samples to confirm the presence of bisphenol-based phenoxy radicals. Impact assessment of transformation products on human health and potential regulations on these alternatives awaits exposure and toxicity analyses conducted with chemically complex mixtures that form from reactions with OH, O_3_, NO_3_, and NO_X_, and UV light.

## Supplementary Material



## Notes

Text: List of chemicals and monitors used; details on offline HRMS analysis and discussion on results; additional details on the PROTEX model; quantification of OH exposure and concentration in the OFR; sample calculation of equivalent exposure time. Tables: Summary of the structure, molecular weight, heating temperature and literature melting point of bisphenols; summary of the observed products; proposed structure of products; proposed structure of radicals and +CO product; summary of the observed products by HPLC-ESI-HRMS analyses; examples of a selection of ring-opening structural isomers of oxidation products; parameters in the PROTEX model for BPA, BPS, and BPAF-related compounds. Figures: schematic diagram of the OFR system; differential positive MS spectrum for BPAF; Compound-specific C_m_, P_ov_ results for BPA, BPS, BPAF, BPB, BPC, BPE, and BPF-related compounds (PDF)
