# Supplementary material for: Multiphase OH Oxidation of Bisphenols: Chemical Transformation and Persistence in the Environment
Source: Environ Sci Technol. 2025 Jun 26;59(26):13319–32. doi: 10.1021/acs.est.5c02030 (PMC12243123; doi:10.1021/acs.est.5c02030)
Supplement: Supplementary file 1 [file es5c02030_si_001.pdf]

Supporting Information for

# Multiphase OH Oxidation of Bisphenols: Chemical Transformation and Persistence in the Environment

Jie Yu,[1] Brandon Wu,[2] Chao Peng,[3] Jeremy Wentzell,[3] Michael J. Wheeler,[3] Joshua O. Osagu,[4] Xianming Zhang,[4] Li Li,[2] Jonathan P.D. Abbatt,[1,*] John Liggio[3,*]

[1] Department of Chemistry, University of Toronto, Toronto, Ontario, M5S 3H6, Canada

[2] School of Public Health, University of Nevada, Reno, Reno, Nevada 89557, United States

[3] Air Quality Research Division, Environment and Climate Change Canada, Toronto, Ontario M3H 5T4, Canada

[4] Department of Chemistry and Biochemistry, Concordia University, Montreal, Quebec H3G 1M8, Canada

***Corresponding authors:**

**John Liggio,** email: John.Liggio@canada.ca

**Jonathan P.D. Abbatt,** e-mail: jonathan.abbatt@utoronto.ca

This document contains:

Pages S1-S24

Text S1- S4

Figure S1-S6

Tables S1-S6

# Text

**Text S1.** List of chemicals and monitors used in the experiment.

Bisphenol A (BPA), Bisphenol S (BPS), Bisphenol F (BPF), Bisphenol B (BPB), Bisphenol C (BPC), Bisphenol E (BPE), Bisphenol AF (BPAF) were purchased from Tokyo Chemical Industry Co., Ltd. (Portland, OR, USA), with purity of at lease 98.0%. Ammonium sulfate (AS), sodium iodide (NaI) and LC-MS grade acetonitrile ($CH_3CN$; ACN) were purchased from Sigma Aldrich Co. (St. Louis, MO, USA). Ultrapure water was obtained from a MilliQ water purification system. $O_3$ was produced by supplying high grade $O_2$ and $N_2$ to an $O_3$ generator (model TG-10; Ozone Solutions).

**Text S2.** Details on offline analysis of the oxidation mixture and discussion on results.

The particles in the flow containing oxidized BPA and BPS were sampled individually using 47 mm Teflon filters (Savillex Inc.) at the OFR exit flow for subsequent HR-ESIMS analyses. For each bisphenol, sample collection lasted for up to 2 days to collect sufficient organic mass (≥ 100 ug). Each filter sample was weighed before and after collection to obtain the total mass collected. An ozone denuder was placed between the OFR exit flow and the filter, to remove $O_3$ at an efficiency of 97% to minimize further oxidation of the sample. A set of control samples was also collected for a similar duration of time when there was only: i) bisphenol, ii) bisphenol and 2 ppm ozone, or iii) bisphenol and UVC light present in the OFR. The filter samples were stored in the dark at - 40 ◦C until extraction. For extraction, each of the filter samples was immersed in 12.5 mL acetonitrile in a 15-mL centrifuge tube and extracted under sonication for 30 min. The extract was then centrifuged for 10 min at 3000 rpm and transferred to a 40 mL vial. The extraction steps were repeated twice, and the two aliquots of the extract were combined. The extracts were analyzed by HPLC-ESI-HRMS, with a LTQ Orbitrap mass spectrometer (Thermal Fisher Scientific, Waltham, MA, USA) equipped with a 1260 Infinity II Preparative LC System (Agilent, Santa Clara, CA, USA). The LC separation was carried out on a reverse phase C-18, Phenomenex column (3.0 µm, 3 × 100 mm) with a mobile phase flow rate at 0.3 mL/min The mobile phase follows a gradient elution with 0.1% formic acid (v/v) in water (A) and 0.1% formic acid (v/v) in acetonitrile (B).  The gradient elution starts with 5% B (0−1 min), increased to 98 % B (1−19 min), held for 3 min (19−21 min), then back to 5% B, and re-equilibrated for 3 minutes was used. The Orbitrap was operated in both positive and negative modes. The mass analyser was utilized in the full scan mode (m/z 100−500, mass resolution 100,000) with data-dependent acquisition. Mass accuracy was within 2 ppm as confirmed with BPA and BPS control samples. The data-processing software Xcalibur was used to extract chromatography and mass spectra of suspected products. A 5ppm mass window was used for searching compounds of interest. In addition to suspected products, a non-targeted workflow (mzmine v3.9) was used to extract all peaks detected with height of greater than 1000 in the extracts.

Discussion of results: As shown in Table S4, most of the $O_3$ control samples (with exposure of 2 ppm $O_3$) also showed the presence of the same products that were observed in OH oxidation samples. We suspect this is likely due to the oxidation of the filter samples by the residual $O_3$ during the offline sampling period. Even though an $O_3$ denuder was used (at a 97% removal efficiency) in front of the filter, the collected mixture was still exposed to a significant amount (~ 60 ppb) of $O_3$ during the long filter collection period. As a result, only 2 oxidation products were detected exclusively in the OH oxidation mixture samples (+2O-4H and +4O). Table S4 summarizes the results for observed products in UVC control and OH oxidation filter samples. However, some products were observed in both type of filter samples; oxidation on the filter during the long collection period cannot be eliminated.

**Text S3.** Additional details on the PROTEX model.

- Compartments and processes considered in the PROTEX model:[1]

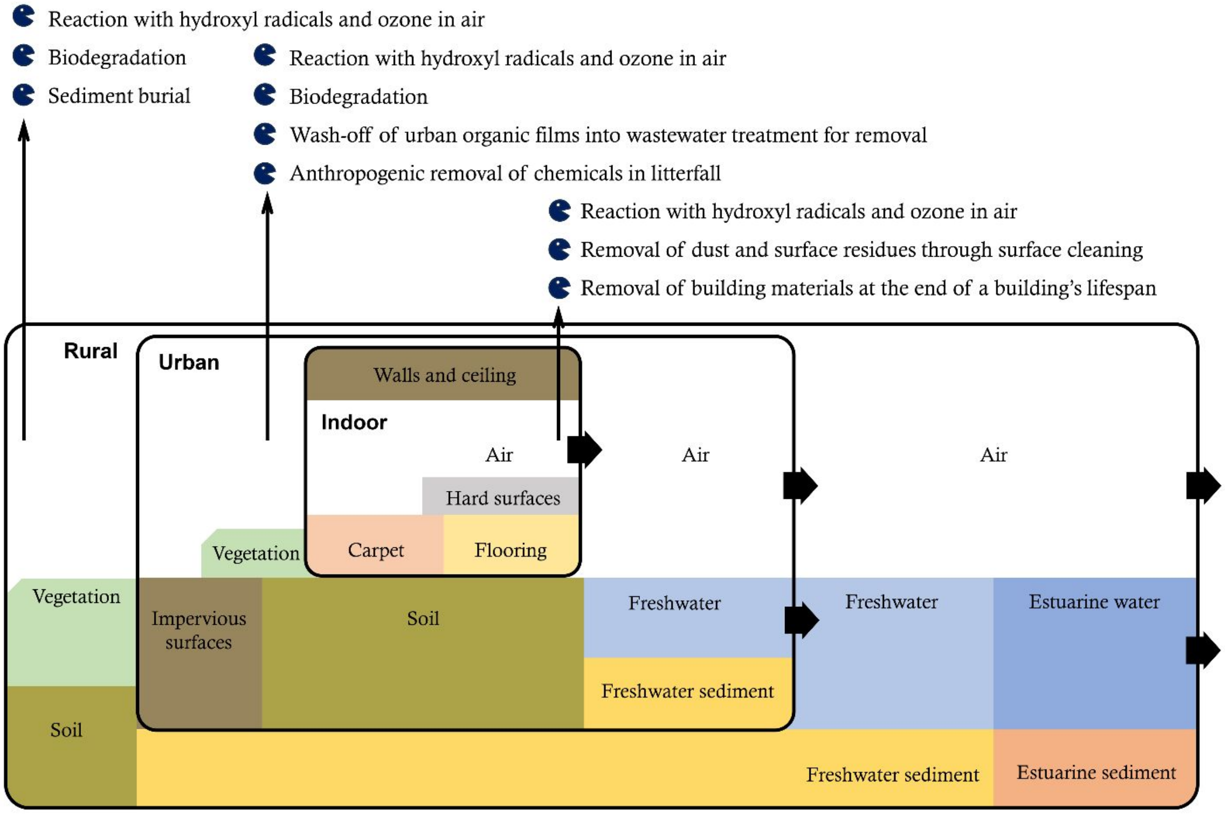


- **Model assumptions.** The model was run under steady-state conditions, assuming constant release of the gas-phase compounds into the indoor air compartment, followed by partitioning with condensed-phase indoor compartments (e.g., carpet, vinyl flooring, hard surfaces, walls and ceilings), removal by gas-phase and surface-bound reactions, human cleaning activities, and transport to urban environments via air exchange.[1]

- **Source of the inputted partitioning and reactivity properties.** The model requires input of partitioning properties (octanol-air and octanol-water partition coefficients; log $K_{OA}$ and log $K_{OW}$, respectively), dissociation constants and reactivity properties (gas-phase reaction rate constants with OH and $O_3$, and biodegradation half-lives; $k_{OH}$, $k_{ozone}$ and $HL_{biodeg}$, respectively) of modeled chemicals. Following the recommended best practice approach as described in Li et al.,[2] these parameters were computed from the identified structures of bisphenols and oxidation products using the "consensus values" of predictions generated by multiple quantitative structure-property relationships (QSPRs), namely, the Estimation Programs Interface (EPI Suite),[3] OPEn Structure-activity/property Relationship App (OPERA),[4] and poly-parameter linear free energy relationships (ppLFERs) with Abraham solute descriptors predicted by the Iterative Fragment Selection (IFS) QSAR [5].

  Adopted from Li et al.,[6] for gas phase kinetic half-life calculations, the concentrations assumed for indoor OH, outdoor OH, indoor $O_3$ and outdoor $O_3$ are $1.7\times 10^5$, $9.7\times 10^5$, $3.5 \times 10^{11}$ and $7.0\times 10^{11}$ molecules $cm^{-3}$, respectively.

- **Surface lifetimes.** Surface lifetimes across a broad range of timescales were applied to represent the complexity in the rates of surface-bound reactions, arising from the kinetic variations associated with the physiochemical properties of the chemical, identity of the oxidant, surface properties, burial depth into the surface, and perturbation from human activities.[7–9]

- **Assumptions on the modeled indoor environment and indoor cleaning efficiency.** As described in Miramontes-Gonzalez and Li (2023),[1] the modeled indoor environment is assumed to be an archetypal "average" American home at 25 ◦C, with an air exchange rate of 0.45 $hour^{-1}$. The floor area is covered by carpet, vinyl flooring and hard surfaces (60%, 40% and 24%, respectively) available. The indoor compartments "walls and ceiling" and "hard surfaces" are assumed to have a 20 nm organic film coating. The above indoor condensed-phase compartments are available for surface partitioning and gas-condensed phase surface reactions. In addition to the heterogeneous surface reactions (modeled by 1, 10 and 100-week surface lifetime scenarios), other indoor loss processes include gas-phase reactions with OH and ozone, dust and surface residue removal by surface cleaning and removal with the building materials at the end of the building's lifespan (100 years) are considered. Cleaning frequencies of twice a month for carpet and flooring and 10 times a month for hard surfaces are assumed, corresponding to half-lives of 250 and 50 h, respectively.[1] In this case, chemicals are mainly partitioning into dust that accumulated on the defined floor area and into "walls and ceilings" compartment (contain 20 nm organic film coating) as surface residue that dissolves in the organic film. The dust bound fraction of the chemicals are regularly removed by cleaning (twice a month or 10 times a month, as described above), whereas the surface-residue fraction is not removed until at the end of the building's lifespan (100 years).[1]

- **Chemical loss processes in the urban and rural environments**. Details are described in Miramontes-Gonzalez and Li (2023).[1]

**Text S4.** Quantification of OH exposure and concentration in the OFR

The OH exposure in the OFR is quantified as the product of the OH concentration and residence time each molecule resides in the OFR. CO was selected as the calibrant given the magnitude of its rate constant with OH. A CO monitor (Model 23r; LGR) was used to measure the CO concentration at the exit of OFR. The reaction of CO with $O_3$ is immeasurably slow at room temperature, and so can be ignored.[10] OH exposure can be determined by the following equation:

$$\text{OH exposure} = -\frac{1}{k_{CO}} \times \ln\frac{[CO]_t}{[CO]_0}$$

where $k_{CO}$ is the rate constant of OH+CO reaction ($k_{CO} = 2.4 \times 10^{-13}$ cm$^3$ molecules$^{-1}$ s$^{-1}$ at 298K, 1 atm),[11] $[CO]_t$ is the CO concentration measured when OH is produced by UV and $O_3$ in OFR, and $[CO]_0$ is when no OH (only UV, no $O_3$) is in the OFR. 2 ppm $O_3$ was supplied to OFR in the presence of 2 UV lamps to generate OH radicals. 2.10 ppm CO ($[CO]_0$) was supplied to OFR in the absence of OH, $[CO]_t$ was measured to be 1.94 ppm under OH oxidation. Thus, the OH exposure was calculated to be $3.3 \times 10^{11}$ molecules s cm$^{-3}$. The residence time of molecules in the OFR is about 120 s, thus the corresponding OH concentration in OFR is $2.8\times10^{9}$ molecules cm$^{-3}$.

**Text S5.** Sample calculation of equivalent exposure time

If the OH concentration in a typical outdoor environment is $1.0\times10^{6}$ molecules cm$^{-3}$, the OH exposure molecules receive in the OFR ($3.3 \times 10^{11}$ molecules s cm$^{-3}$) is equivalent to about 4 days outdoors. The equivalent outdoor exposure time can be calculated as below:

$$\text{Exposure Time} = \frac{OH\ exposure\ in\ OFR}{OH\ concentration} = \frac{3.3 \times 10^{11} molecules\ s\ cm^{-3}}{1.0\times 10^{6} molecules\ cm^{-3}} = 3.3\times10^{5}\ \text{s} \approx \underline{\text{4 days}}$$

# Table

**Table S1.** Summary of the structure, molecular weight, heating temperature and literature melting point of bisphenols.

| **Compound Name & CAS number** | **Structure** | **Molecular Weight (g/mol)** | **Heating Tempeature (◦C)** | **Literature Melting Point(◦C)** |
|---|---|---|---|---|
| BPA 80-05-7 | 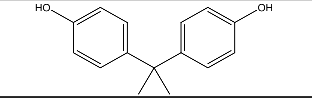 | 228.29 | 120 | 160 [1] |
| BPS 80-09-1 | 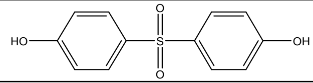 | 250.27 | 180 | 241 [1] |
| BPF 620-92-8 | 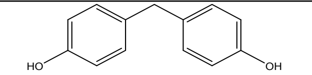 | 200.23 | 135 | 163 [1] |
| BPB 77-40-7 | 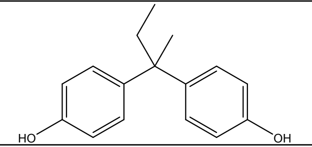 | 242.31 | 108 | 121 [1] |
| BPC 14868-03-2 | 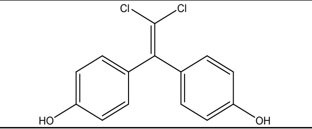 | 281.13 | 165 | 213 [2] |
| BPE 2081-08-5 | 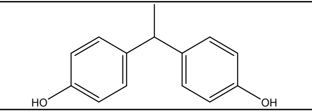 | 214.26 | 140 | 160 [3] |
| BPAF 1478-61-1 | 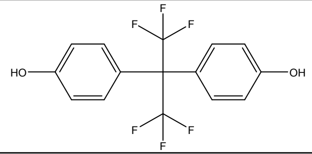 | 336.23 | 155 | 159 [1] |

[1] Melting point value was obtained from the PubChem database provided by the National Library of Medicine.

[2] Melting point value was obtained from the ChemSpider database provided by the Royal Sociaty of Chemistry.

[3] Melting point value was obtained from the safety data sheet provided by Sigma Aldrich Co.

**Table S2.** Summary of **(a)** the observed transformation products (excluding radicals and +CO product) by EESI-TOFMS, and **(b)** the proposed structure of each product for a bisphenol (BPX) that matches with the label in the mechanism (Figure 3). A checkmark in (a) indicates an observed product formula. The corresponding BPX label used in PROTEX model analyses was also provided.

**(a)**

| | +1O | | +2O | | | | +3O | | | | +4O | | +5O | |
|---|---|---|---|---|---|---|---|---|---|---|---|---|---|---|
| Label in the mechanism (Figure 3) | P10 | P1 | P7 | P6 | P2 | P11 | P9 | P8 | P3 | P12 | P4 | P13 | P5 | P14 |
| Product | +1O -2H | +1O | +2O -4H | +2O -2H | +2O | +2O +2H | +3O -4H | +3O -2H | +3O | +3O +2H | +4O | +4O +2H | +5O | +5O +2H |
| BPA | √ | √ | √ | √ | √ | | | √ | √ | | √ | | | |
| BPS | √ | √ | | √ | √ | √ | | √ | √ | √ | | | | |
| BPF | √ | √ | √ | √ | √ | √ | √ | √ | √ | √ | √ | √ | | |
| BPB | √ | √ | √ | √ | √ | | | | √ | | √ | | | |
| BPC | | √ | | √ | | | | | | | √ | | | |
| BPE | √ | √ | √ | √ | √ | | | √ | | √ | | | | |
| BPAF | √ | | √ | √ | √ | | √ | √ | √ | √ | √ | √ | √ | √ |

**(b)**

| Compound | Label in mechanism (Figure 3) | Label in PROTEX analysis | Location in Figure 3 | Structure |
|---|---|---|---|---|
| Parent | BPX | BPX00 | Panel A | 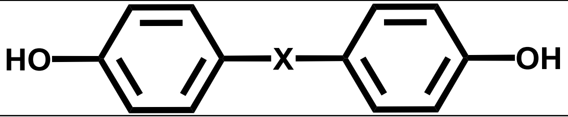  |
| +1O | P1 | BPX01 | Pathway A; Panel A | 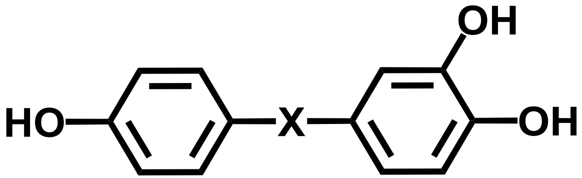  |

| | | | | |
|---|---|---|---|---|
| +2O | P2 | BPX02 | Pathway A; Panel A | 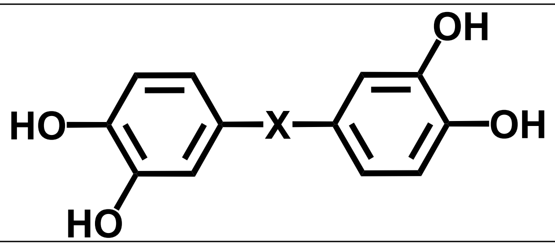 |
| +3O | P3 | BPX03 | Pathway A; Panel A | 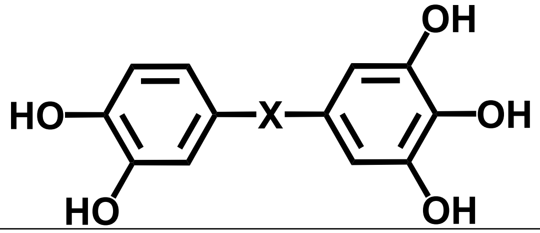 |
| +4O | P4 | BPX04 | Pathway A; Panel A | 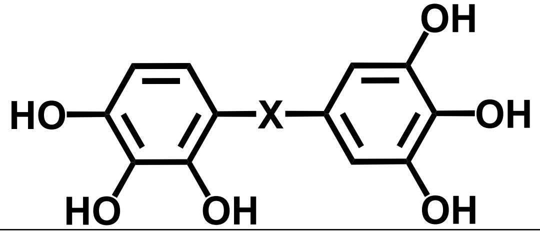 |
| +5O | P5 | BPX05 | Pathway A; Panel A | 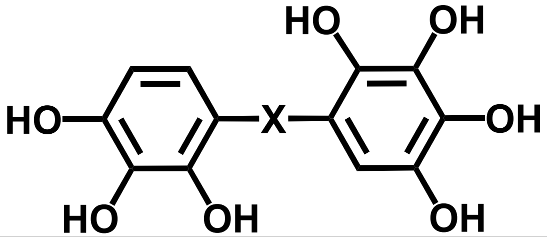 |
| +2O-2H | P6 | BPX06 | Pathway A; Panel A | 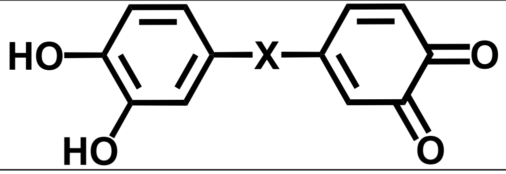 |

| | | | | |
|---|---|---|---|---|
| +2O-4H | P7 | BPX07 | Pathway A; Panel A | 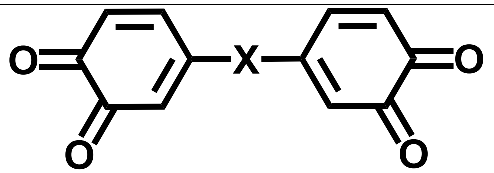 |
| +3O-2H | P8 | BPX08 | Pathway A; Panel A | 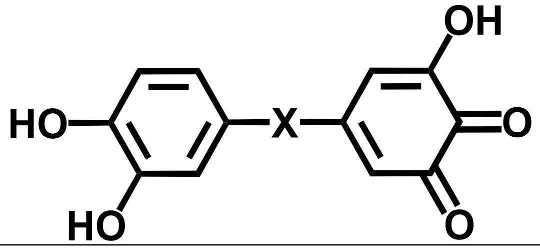 |
| +3O-4H | P9 | BPX09 | Pathway A; Panel A | 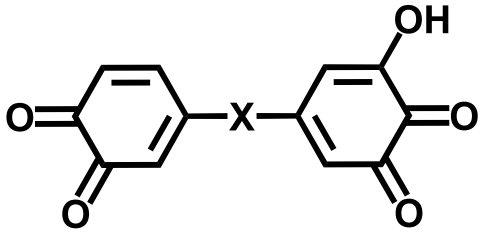 |
| +1O-2H | P10 | BPX10 | Pathway B; Panel A | 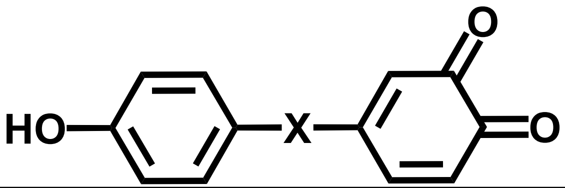 |
| +2O+2H | P11 | BPX11 | Pathway D; Panel B | 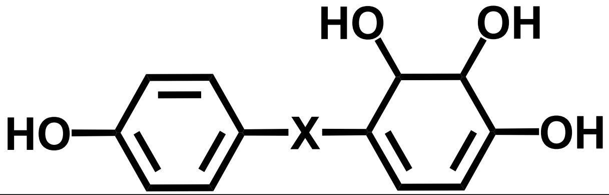 |
| +3O+2H | P12 | BPX12 | Pathway E; Panel B | 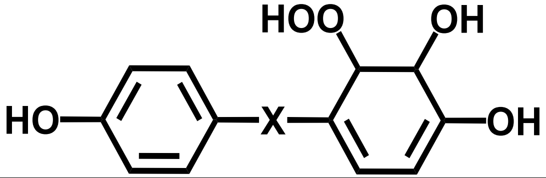 |

| | | | | |
|---|---|---|---|---|
| +4O+2H | P13 | BPX13 | Pathway D; Panel B | 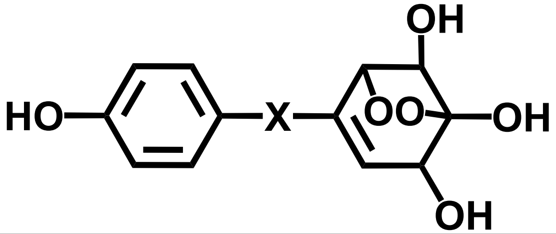 |
| +5O+2H | P14 | BPX14 | Pathway E; Panel B | 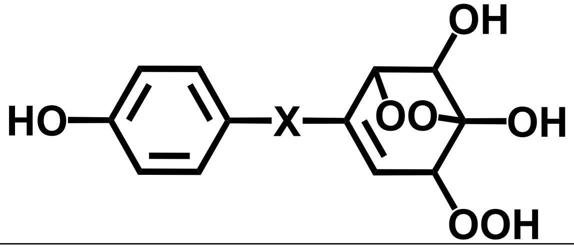 |
| +1O | P1.1 | / | Pathway B; Panel A | 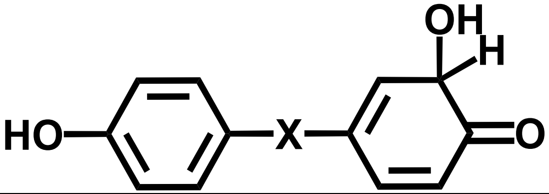 |
| +1O | P1.2 | / | Pathway C; Panel B | 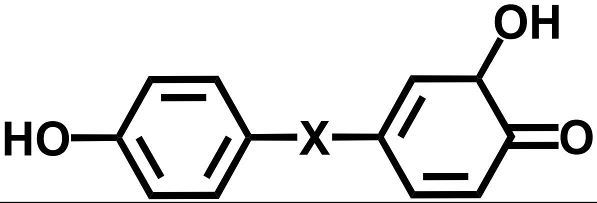 |
| +2O | P2.1 | / | Pathway B; Panel A | 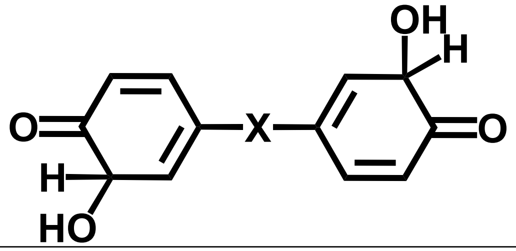 |

| | | | | |
|---|---|---|---|---|
| +2O | P2.2 | / | Pathway D; Panel B | 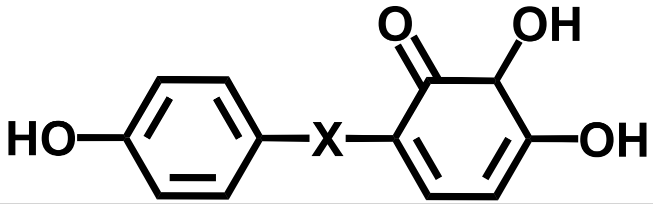 |
| +4O | P4.1 | / | Pathway D; Panel B | 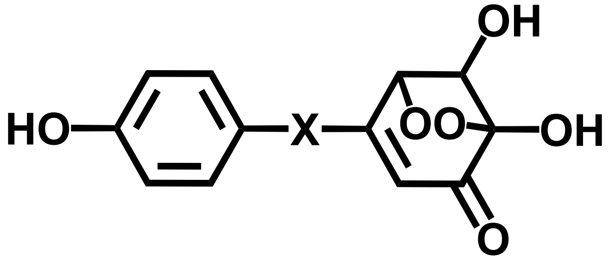 |
| +2O-2H | P6.1 | / | Pathway B; Panel A | 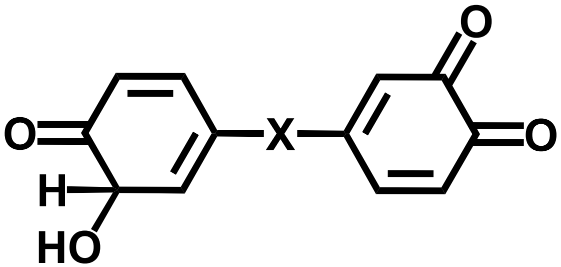 |
| +2O-4H | P7.1 | / | Pathway B; Panel A | 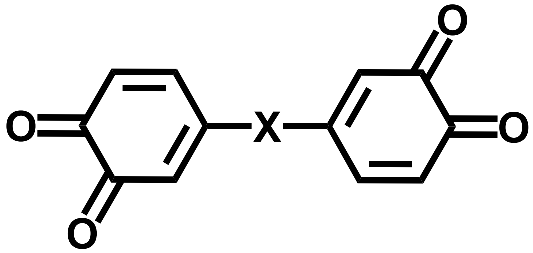 |

**Table S3. (a)** Proposed structure of radicals and +CO product and the corresponding labels in mechanism (Figure 3). **(b)** Summary of the observed radical and +CO products by EESI-TOFMS.

**(a)**

| Product | Label in mechanism (Figure 3) | Structure |
|---|---|---|
| +1O-1H | PR1 | 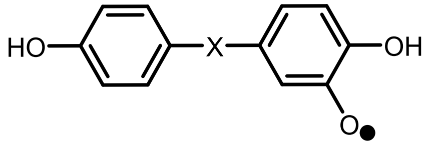 |
| +2O-1H | PR2 | 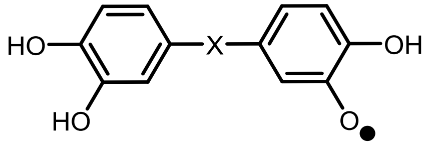 |
| +3O-1H | PR3 | 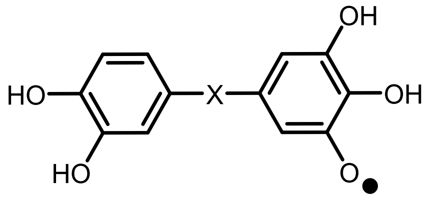 |
| +1O-3H | PR4 | 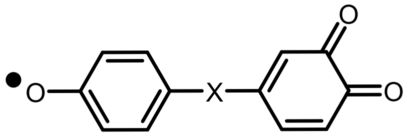 |
| +CO | / | 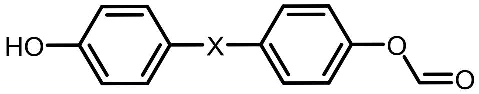 |

**(b)**

| Label in the mechanism (Figure 3) | PR1 | PR4 | PR2 | PR3 | / |
|---|---|---|---|---|---|
| Product | +1O -1H | +1O-3H | +2O -1H | +3O -1H | +CO |
| BPA | √ | | √ | | √ |
| BPS | √ | | | | |
| BPF | √ | | √ | | √ |
| BPB | √ | | | | √ |
| BPC | | | √ | | √ |
| BPE | √ | √ | | | √ |
| BPAF | | | √ | √ | √ |

**Table S4.** Summary of the observed products (excluding radicals and +CO product) by HPLC-ESI-HRMS analyses of offline filter samples for BPA and BPS oxidation. A checkmark indicates an observed product formula. Check marks in red indicate the products only observed with OH exposure. Note that the results for ozone only control experiments were not included here due to filter samples' long-term exposure to the residual ozone during the collection period. Previous work by Yu et al. has provided a series of products and proposed mechanisms for the surface-bound ozonolysis of BPA under indoor-relevant conditions.[12]

| | | +1O | | +2O | | | | +3O | | | | +4O | | +5O | |
|---|---|---|---|---|---|---|---|---|---|---|---|---|---|---|---|
| | Label in the mechanism (Figure 3) | P10 | P01 | P7 | P6 | P2 | P11 | P9 | P8 | P3 | P12 | P04 | P13 | P5 | P14 |
| | Product | +1O -2H | +1O | +2O -4H | +2O -2H | +2O | +2O +2H | +3O -4H | +3O -2H | +3O | +3O +2H | +4O | +4O +2H | +5O | +5O +2H |
| BPA | UVC only (control) | | √ | | | | √ | √ | | | √ | | √ | √ | |
| | OH (with residual $O_3$) | √ (red) | √ | √ (red) | √ (red) | √ (red) | | | √ (red) | √ (red) | | √ (red) | √ | √ | |
| BPS | UVC only (control) | | √ | | √ | | | √ | √ | | | | √ | √ | √ |
| | OH (with residual $O_3$) | | √ | | | | | √ | √ | | √ (red) | √ (red) | √ | √ | |

**Table S5.** Examples of a selection of ring-opening structural isomers of oxidation products presented in Figure 3. These structures are proposed based on previous studies on OH oxidation of bisphenols and phenolic compounds in aqueous phase.[13–15]

| Product | Structure | Source |
| --- | --- | --- |
| +3O | 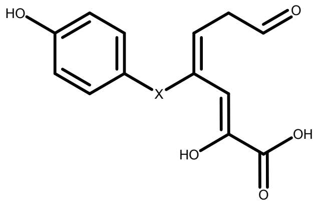 | Wang et al. (2022)[13] |
| +3O | 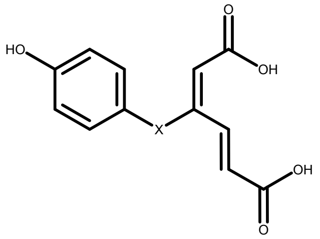 | Hems et al. (2018)[14] and Procar-Santos et al. (2022)[15] |
| +4O | 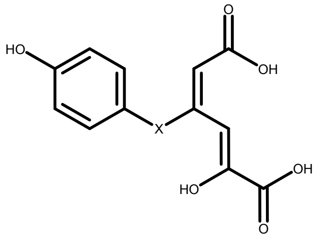 | Hems et al. (2018)[14] |
| +5O | 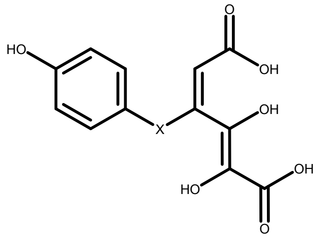 | Hems et al. (2018)[14] |

**Table S6.** Partition coefficients and reactivity properties in the PROTEX model for BPA, BPS and BPAF-related compounds: (a) octanol–water and octanol–air partition coefficients (log $K_{OA}$ and log $K_{OW}$); **(b)** acid and base dissociation constant (p$K_{a,a}$ and p$K_{a,b}$); gas-phase reactions rates with OH and $O_3$ ($k_{OH}$ and $k_{O3}$); **(c)** biodegradation half-life ($HL_{biodegradation}$) and the result of overall outdoor persistence ($P_{ov,o}$); Partition coefficients are the consensus values (geometric means) obtained from the predictions from computational tools, including Estimation Programs Interface (EPI Suite),[3] OPEn Structure-activity/property Relationship App (OPERA),[4] and poly-parameter linear free energy relationships (ppLFERs) with Abraham solute descriptors predicted by the Iterative Fragment Selection (IFS) QSAR.[5]

**(a)** log $K_{OA}$ and log $K_{OW}$

| | BPA | | BPS | | BPAF | |
|---|---|---|---|---|---|---|
| Compound Label (as in Figure 3) | log $K_{OA}$ | log $K_{OW}$ | log $K_{OA}$ | log $K_{OW}$ | log $K_{OA}$ | log $K_{OW}$ |
| Parent | 11.6 | 3.5 | 13.1 | 1.2 | 11.1 | 3.7 |
| P1; +1O | 13.5 | 3.3 | 14.8 | 1.4 | 12.7 | 3.6 |
| P2; +2O | 15.2 | 2.9 | 16.5 | 1.5 | 14.4 | 3.4 |
| P3; +3O | 17.3 | 2.0 | 18.7 | 1.3 | 16.8 | 3.0 |
| P4; +4O | 19.4 | 1.7 | 20.9 | 1.1 | 18.9 | 2.7 |
| P5; +5O | 24.2 | 1.1 | 25.5 | 0.9 | 23.7 | 2.0 |
| P6; +2O-2H | 13.3 | 2.2 | 14.0 | -0.4 | 12.5 | 2.9 |
| P7; +2O-4H | 11.0 | 1.2 | 11.8 | -1.1 | 10.4 | 2.2 |
| P8; +3O-2H | 15.4 | 1.6 | 16.1 | -0.4 | 14.9 | 2.3 |
| P9; +3O-4H | 11.8 | 0.1 | 12.7 | -2.3 | 11.4 | 1.2 |
| P10; +1O-2H | 12.1 | 2.8 | 12.8 | -0.01 | 11.3 | 3.5 |
| P11; +2O+2H | 11.8 | 2.3 | 12.3 | -0.2 | 11.0 | 3.2 |
| P12; +3O+2H | 14.1 | 1.6 | 14.5 | -0.6 | 13.3 | 2.7 |
| P13; +4O+2H | 13.9 | -0.3 | 14.4 | -2.5 | 13.4 | 0.02 |
| P14+5O+2H | 14.9 | -0.8 | 15.4 | -2.8 | 14.3 | -0.2 |

**(b)** $pK_{a,a}$, $pK_{a,b}$, $k_{OH}$ and $k_{O3}$

| | **BPA** | | | | **BPS** | | | | **BPAF** | | | |
|---|---|---|---|---|---|---|---|---|---|---|---|---|
| Compound Label (as in Figure 3) | $pK_{a,a}$ | $pK_{a,b}$ | $k_{OH}$ (cm³/molecule s) | $k_{O3}$ (cm³/molecule s) | $pK_{a,a}$ | $pK_{a,b}$ | $k_{OH}$ (cm³/molecule s) | $k_{O3}$ (cm³/molecule s) | $pK_{a,a}$ | $pK_{a,b}$ | $k_{OH}$ (cm³/molecule s) | $k_{O3}$ (cm³/molecule s) |
| Parent | 9.5 | / | $4.8\times10^{-11}$ | / | 7.0 | / | $1.1\times10^{-11}$ | / | 8.2 | / | $5.0\times10^{-11}$ | / |
| P1; +1O | 9.2 | / | $5.4\times10^{-11}$ | / | 7.0 | / | $1.2\times10^{-11}$ | / | 7.9 | / | $5.1\times10^{-11}$ | / |
| P2; +2O | 8.8 | / | $5.9\times10^{-11}$ | / | 6.8 | / | $1.1\times10^{-11}$ | / | 7.5 | / | $5.5\times10^{-11}$ | / |
| P3; +3O | 8.2 | / | $1.1\times10^{-10}$ | / | 6.5 | / | $5.3\times10^{-11}$ | / | 6.9 | / | $1.1\times10^{-10}$ | / |
| P4; +4O | 8.4 | / | $1.1\times10^{-10}$ | / | 6.2 | / | $9.2\times10^{-11}$ | / | 6.8 | / | $1.1\times10^{-10}$ | / |
| P5; +5O | 8.7 | / | $1.1\times10^{-10}$ | / | 5.8 | / | $7.9\times10^{-11}$ | / | / | 3.96 | $1.0\times10^{-10}$ | / |
| P6; +2O-2H | 9.5 | / | $4.6\times10^{-11}$ | $8.1\times10^{-18}$ | 7.0 | / | $1.9\times10^{-11}$ | $8.1\times10^{-18}$ | 8.4 | / | $4.5\times10^{-11}$ | $8.1\times10^{-18}$ |
| P7; +2O-4H | / | / | $2.9\times10^{-11}$ | $1.6\times10^{-17}$ | / | / | $3.0\times10^{-11}$ | $1.6\times10^{-17}$ | / | / | $3.1\times10^{-11}$ | $1.6\times10^{-17}$ |
| P8; +3O-2H | 8.9 | / | $1.2\times10^{-10}$ | $8.1\times10^{-18}$ | 6.5 | / | $5.9\times10^{-11}$ | $8.1\times10^{-18}$ | 7.8 | / | $1.2\times10^{-10}$ | $8.1\times10^{-18}$ |
| P9; +3O-4H | / | / | $3.2\times10^{-11}$ | $1.6\times10^{-17}$ | / | / | $3.2\times10^{-11}$ | $1.6\times10^{-17}$ | / | / | $3.6\times10^{-11}$ | $1.6\times10^{-17}$ |
| P10; +1O-2H | 10.3 | / | $4.4\times10^{-11}$ | $8.1\times10^{-18}$ | 7.4 | / | $2.0\times10^{-11}$ | $8.1\times10^{-18}$ | 9.3 | / | $3.7\times10^{-11}$ | $8.1\times10^{-18}$ |
| P11; +2O+2H | 8.6 | / | $1.8\times10^{-10}$ | $3.4\times10^{-16}$ | 8.4 | / | $1.8\times10^{-10}$ | $3.4\times10^{-16}$ | 7.6 | / | $1.8\times10^{-10}$ | $3.4\times10^{-16}$ |
| P12; +3O+2H | 12.6 | 4.71 | $1.8\times10^{-10}$ | $3.4\times10^{-16}$ | 10.5 | / | $1.8\times10^{-10}$ | $3.4\times10^{-16}$ | 11.4 | / | $1.7\times10^{-10}$ | $3.4\times10^{-16}$ |
| P13; +4O+2H | 6.6 | / | $1\times10^{-10}$ | $7.4\times10^{-17}$ | 7.2 | / | $8.7\times10^{-11}$ | $7.4\times10^{-17}$ | 6.4 | / | $9.7\times10^{-11}$ | $7.4\times10^{-17}$ |
| P14+5O+2H | 7.6 | / | $9.3\times10^{-11}$ | $7.4\times10^{-17}$ | 7.4 | / | $8.0\times10^{-11}$ | $7.4\times10^{-17}$ | 7.5 | / | $9.0\times10^{-11}$ | $7.4\times10^{-17}$ |

**(c)** $HL_{biodegradation}$ and $P_{ov,o}$

| | BPA | | BPS | | BPAF | |
|---|---|---|---|---|---|---|
| Compound Label (as in Figure 3) | $HL_{biodegradation}$ (hour) | $P_{ov,o}$ (hour) | $HL_{biodegradation}$ (hour) | $P_{ov,o}$ (hour) | $HL_{biodegradation}$ (hour) | $P_{ov,o}$ (hour) |
| Parent | $6.9\times10^{2}$ | $6.2\times10^{3}$ | $5.7\times10^{2}$ | $4.8\times10^{3}$ | $7.9\times10^{2}$ | $1.9\times10^{4}$ |
| P1; +1O | $6.1\times10^{2}$ | $5.2\times10^{3}$ | $5.1\times10^{2}$ | $4.1\times10^{3}$ | $7.0\times10^{2}$ | $1.8\times10^{4}$ |
| P2; +2O | $5.5\times10^{2}$ | $4.4\times10^{3}$ | $4.5\times10^{2}$ | $3.6\times10^{3}$ | $6.2\times10^{2}$ | $1.6\times10^{4}$ |
| P3; +3O | $4.9\times10^{2}$ | $3.7\times10^{3}$ | $4.0\times10^{2}$ | $3.1\times10^{3}$ | $5.5\times10^{2}$ | $1.5\times10^{4}$ |
| P4; +4O | $4.3\times10^{2}$ | $3.2\times10^{3}$ | $3.6\times10^{2}$ | $2.7\times10^{3}$ | $4.9\times10^{2}$ | $1.3\times10^{4}$ |
| P5; +5O | $3.8\times10^{2}$ | $2.7\times10^{3}$ | $3.2\times10^{2}$ | $2.3\times10^{3}$ | $4.4\times10^{3}$ | $1.2\times10^{4}$ |
| P6; +2O-2H | $9.0\times10^{2}$ | $6.4\times10^{3}$ | $7.0\times10^{2}$ | $5.4\times10^{3}$ | $9.7\times10^{3}$ | $1.9\times10^{4}$ |
| P7; +2O-4H | $1.3\times10^{3}$ | $9.4\times10^{3}$ | $1.1\times10^{3}$ | $8.1\times10^{3}$ | $1.5\times10^{3}$ | $2.2\times10^{4}$ |
| P8; +3O-2H | $7.6\times10^{2}$ | $5.5\times10^{3}$ | $6.2\times10^{2}$ | $4.7\times10^{3}$ | $8.6\times10^{3}$ | $1.7\times10^{4}$ |
| P9; +3O-4H | $9.0\times10^{2}$ | $7.0\times10^{3}$ | $7.4\times10^{2}$ | $5.8\times10^{3}$ | $1.0\times10^{4}$ | $1.9\times10^{4}$ |
| P10; +1O-2H | $9.6\times10^{2}$ | $7.4\times10^{3}$ | $7.9\times10^{2}$ | $6.2\times10^{3}$ | $1.0\times10^{4}$ | $2.0\times10^{4}$ |
| P11; +2O+2H | $2.4\times10^{2}$ | $2.4\times10^{3}$ | $2.0\times10^{2}$ | $1.9\times10^{3}$ | $2.7\times10^{3}$ | $1.4\times10^{4}$ |
| P12; +3O+2H | $4.3\times10^{2}$ | $3.6\times10^{3}$ | $3.6\times10^{2}$ | $3.0\times10^{3}$ | $4.9\times10^{3}$ | $1.5\times10^{4}$ |
| P13; +4O+2H | $6.5\times10^{2}$ | $5.1\times10^{3}$ | $5.4\times10^{2}$ | $4.3\times10^{3}$ | $7.5\times10^{3}$ | $1.7\times10^{4}$ |
| P14+5O+2H | $1.2\times10^{3}$ | $7.6\times10^{3}$ | $9.8\times10^{2}$ | $6.6\times10^{3}$ | $1.4\times10^{4}$ | $1.8\times10^{4}$ |

# Figures

**Figure S1.** Schematic diagram of the oxidation flow reactor (OFR) system. $O_3$ mixing ratio in OFR is maintained at 2 ppm at 45% RH. With UV radiation, the OH exposure is approximately $3.3 \times 10^{11}$ molecules s $cm^{-3}$.

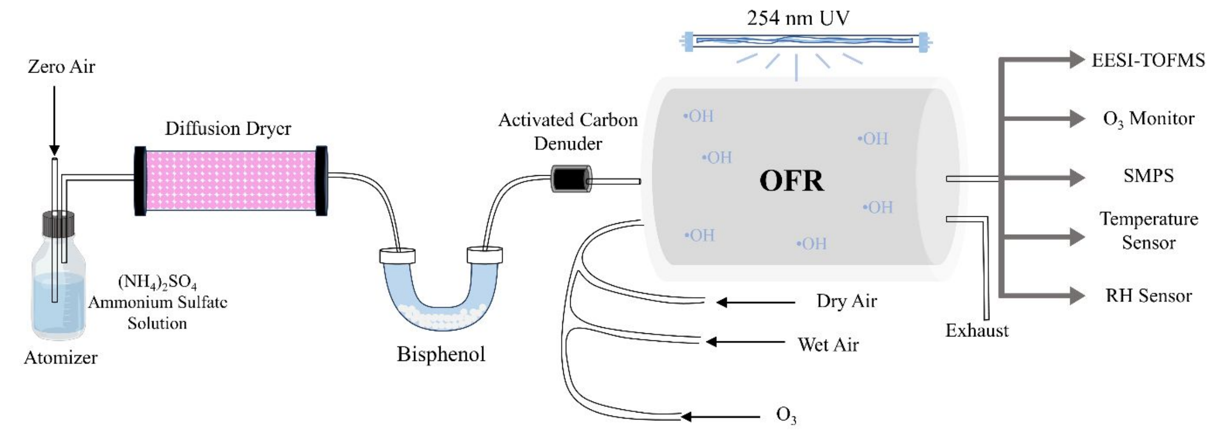


**Figure S2.** Differential positive EESI-TOFMS spectrum for BPAF at OH exposure of $3.3 \times 10^{11}$ molecules s $cm^{-3}$. Only a minimal change was observed for the weak parent ion BPAF (*m/z* 359). This is likely due to two reasons: the low solubility of BPAF in the working solution and a slightly increasing parent ion signal during the course of the experiment.

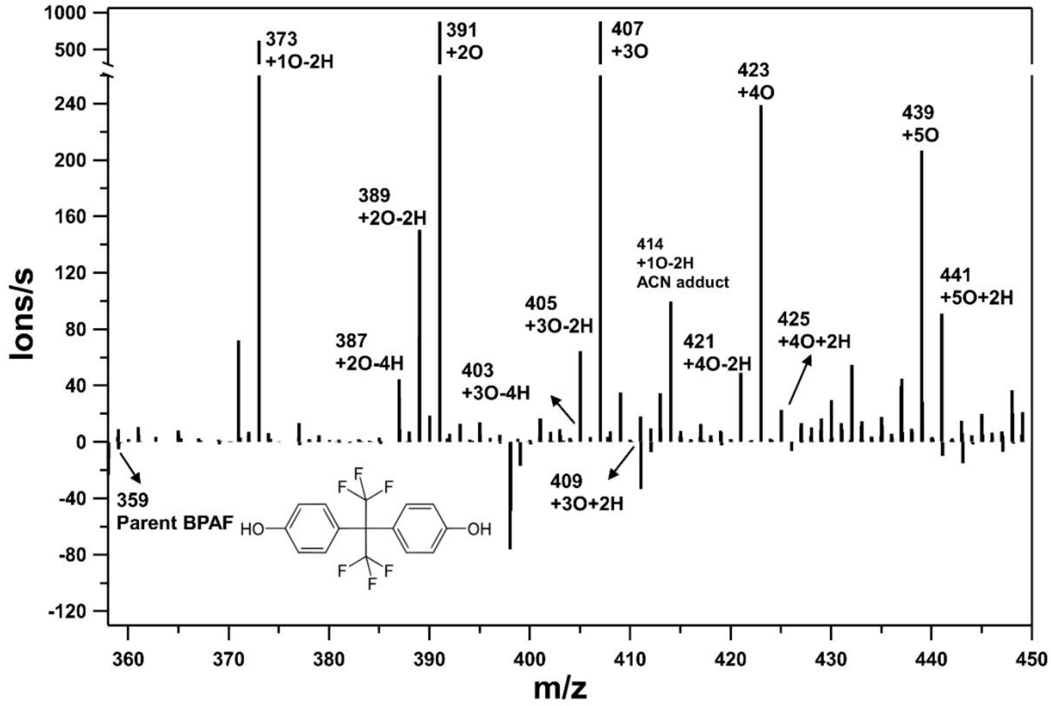

**Figure S3.** Compound-specific indoor chemical mass distribution ($C_m$) for BPA, BPS and BPAF-related compounds. The top, middle and bottom panels show results for the surface lifetime assumption of 1, 10, and 100-weeks, respectively. BPX is the generalized labeling for bisphenols. The parent bisphenols are labelled as "BPX00" and the rest of the numerical labels correspond to the transformation products provided in the mechanism (Figure 3), i.e. P1 in Figure 3 corresponds to BPX01 here. The $C_m$ results for other bisphenols (BPB, C, E and F) are provided in Figure S5.

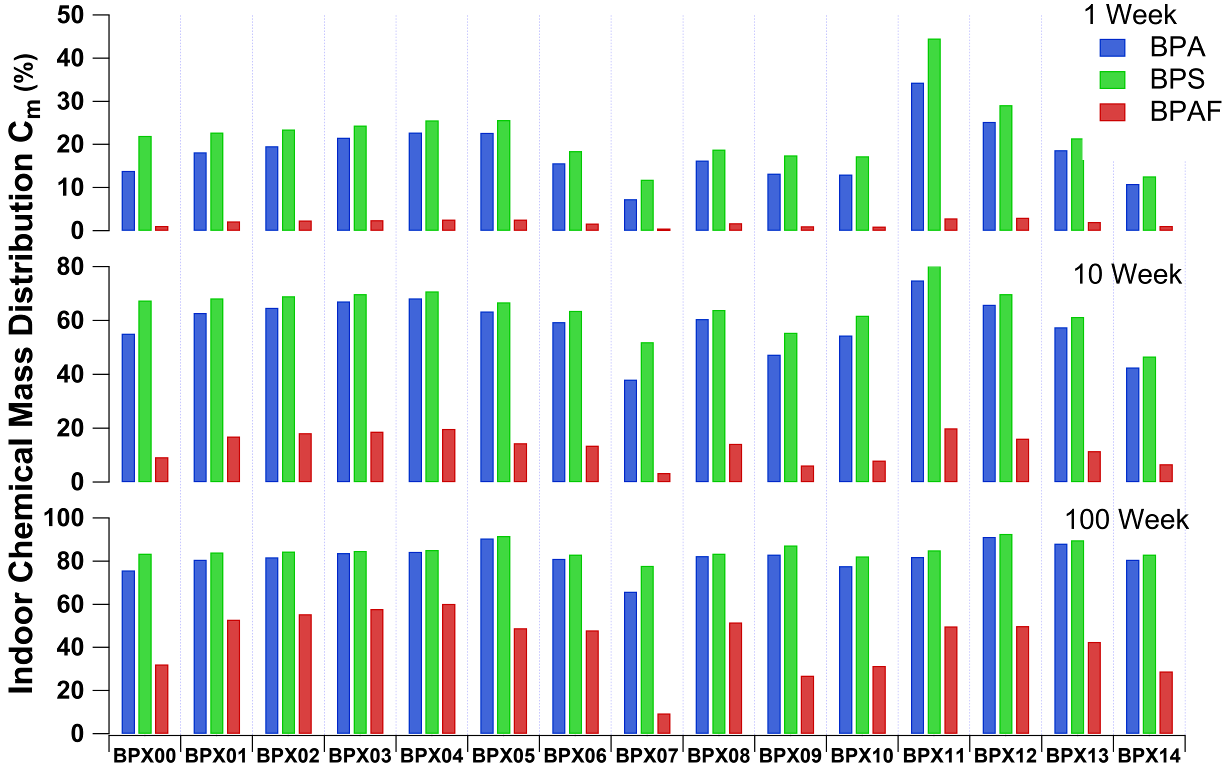

**Figure S4.** Compound-specific indoor and outdoor overall persistence ($P_{ov,i}$ and $P_{ov,o}$) for BPA, BPS and BPAF-related compounds. The top, middle and bottom panels show results under the assumption of 1, 10, and 100-week surface lifetime, respectively. Solid bars represent indoor persistence ($P_{ov,i}$), whereas dashed bars are for persistence in the outdoor (rural) environment ($P_{ov,o}$). BPX is the generalized labeling for bisphenols. The parent bisphenols are labelled as "BPX00" and the rest of the numerical labels correspond to the transformation products provided in the mechanism (Figure 3), i.e., P1 in Figure 3 corresponds to BPX01 here. The $P_{ov}$ results for other bisphenols (BPB, C, E and F) are provided in Figure S6.

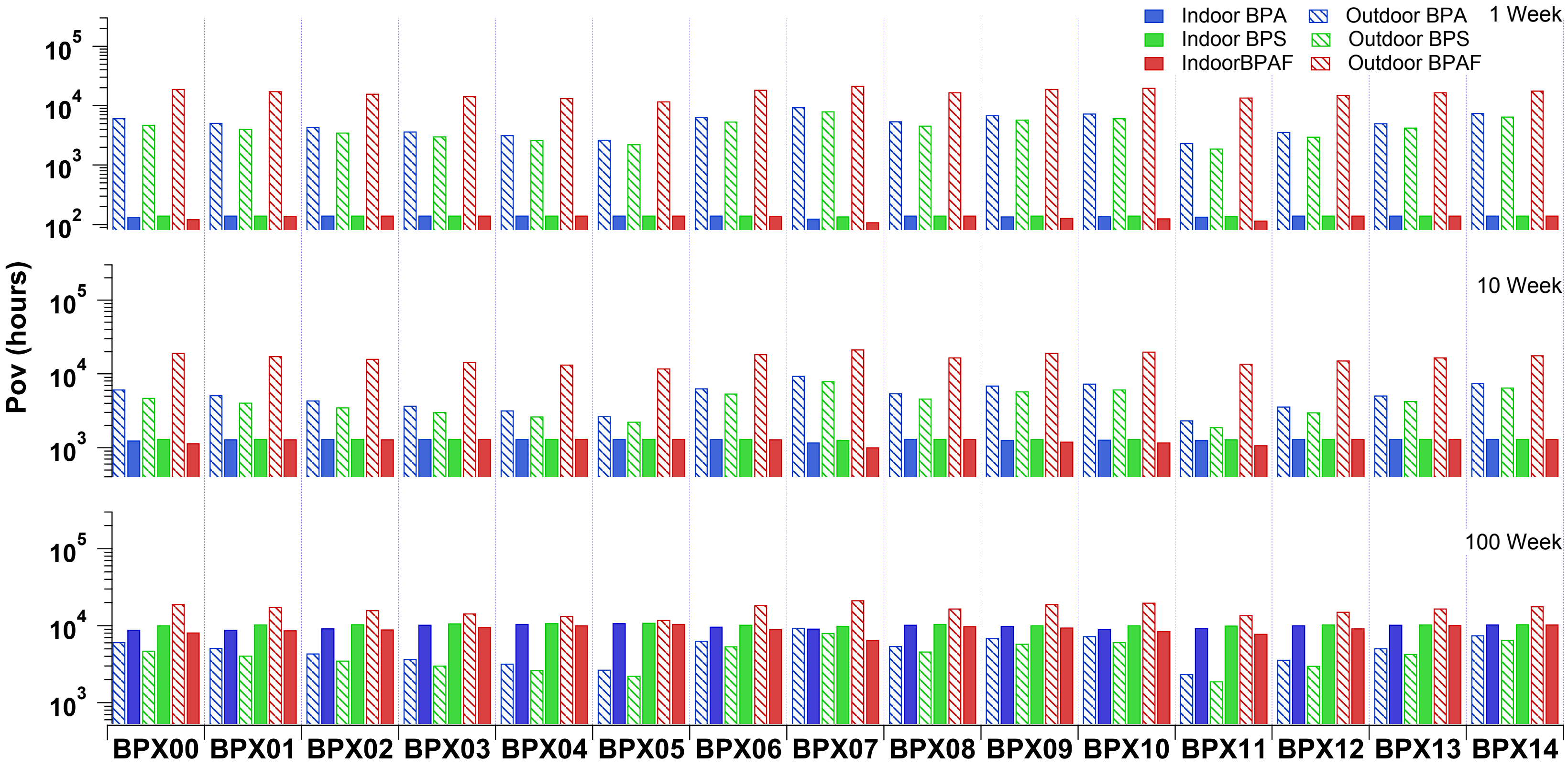

**Figure S5. (a)** Indoor chemical mass distribution ($C_m$) results for BPB, BPC, BPE and BPF-related compounds. The $C_m$ results obtained under the assumption of 1, 10 and 100-week surface lifetimes are shown in black, blue and red circles, respectively. The $C_m$ values for parent bisphenols are presented in solid circles, whereas the closed-shell products are presented in hollow circles. **(b)** Compound-specific $C_m$ for BPA, BPS and BPAF-related compounds. The top, middle and bottom panels show results for the surface lifetime assumption of 1, 10, and 100-weeks, respectively. BPX is the generalized labeling for bisphenols. The parent bisphenols are labelled as "BPX00" and the rest of the numerical labels correspond to the transformation products provided in the mechanism (Figure 3), i.e. P1 in Figure 3 corresponds to BPX01 here.

**(a)**

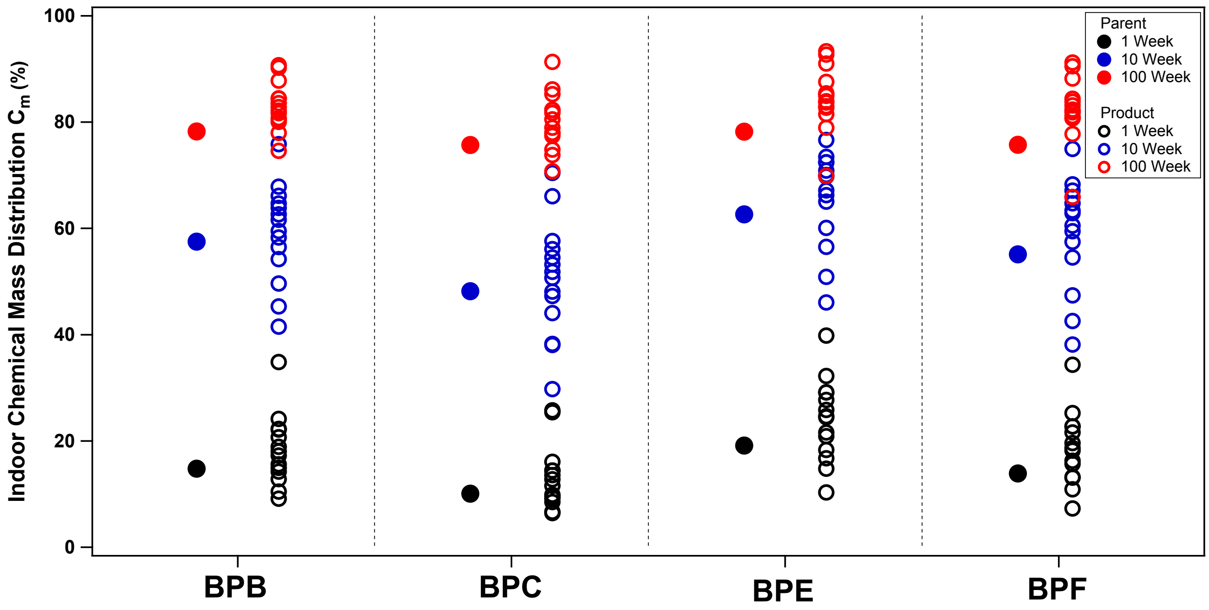

**(b)**

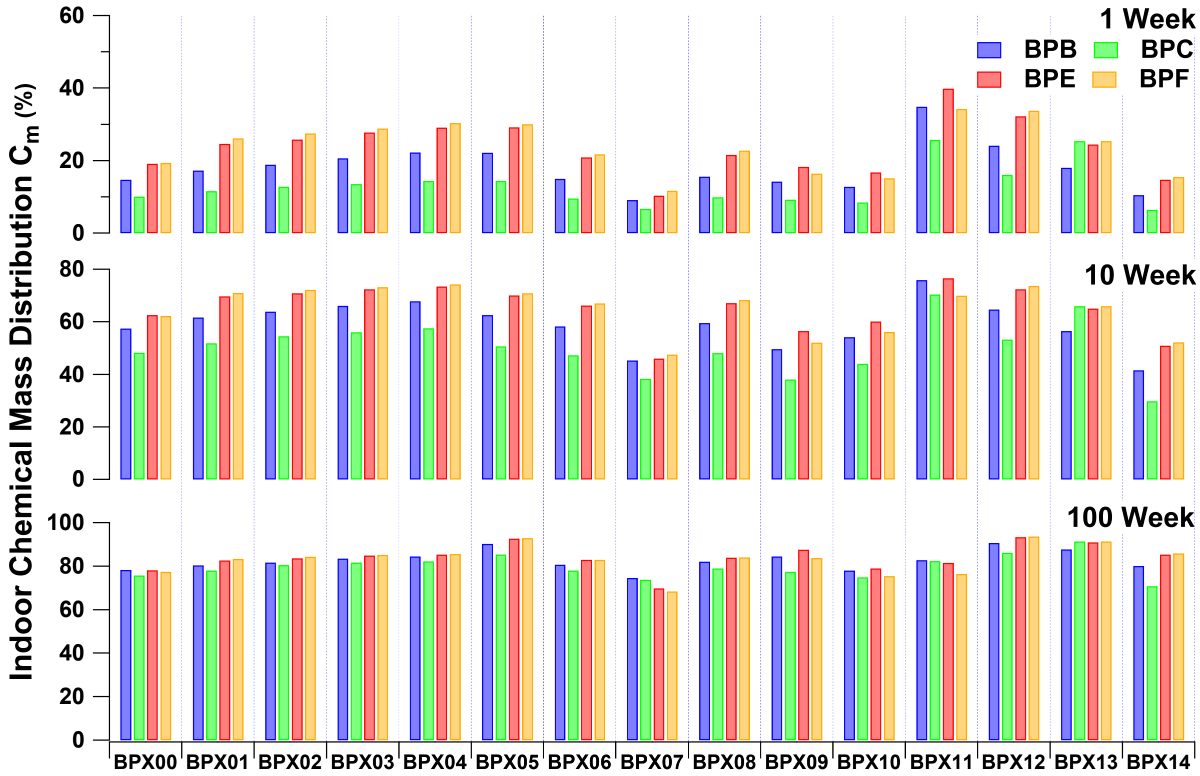
Indoor Chemical Mass Distribution $C_m$ (%)
1 Week
10 Week
100 Week
BPB
BPC
BPE
BPF
BPX00
BPX01
BPX02
BPX03
BPX04
BPX05
BPX06
BPX07
BPX08
BPX09
BPX10
BPX11
BPX12
BPX13
BPX14

**Figure S6. (a)** Indoor and Outdoor overall persistence ($P_{ov,i}$ and $P_{ov,o}$) for BPB, BPC, BPE and BPF-related compounds. The $P_{ov,i}$ results obtained under the assumption of 1, 10 and 100-week surface lifetimes are shown in black, blue and red circles, respectively. The $P_{ov,i}$ values for parent bisphenols are presented in solid circles, whereas the neutral products are presented in hollow circles. The $P_{ov,o}$ values for parent bisphenols and neutral products are presented in solid and hollow green stars, respectively. **(b)** Compound-specific $P_{ov,i}$ and $P_{ov,o}$ for BPB, BPC, BPE and BPF-related compounds. The top, middle and bottom panels show results for the surface lifetime assumption of 1, 10, and 100-weeks, respectively. BPX is the generalized labeling for bisphenols. The parent bisphenols are labelled as “BPX00” and the rest of the numerical labels correspond to the transformation products provided in the mechanism (Figure 3), i.e. P1 in Figure 3 corresponds to BPX01 here.

**(a)**

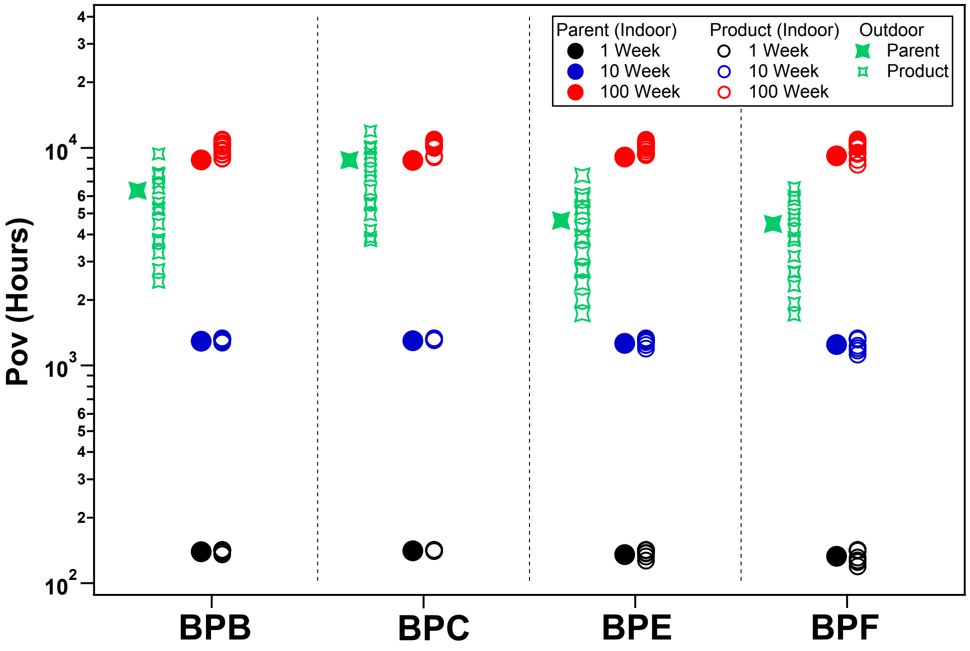

241 **(b)**

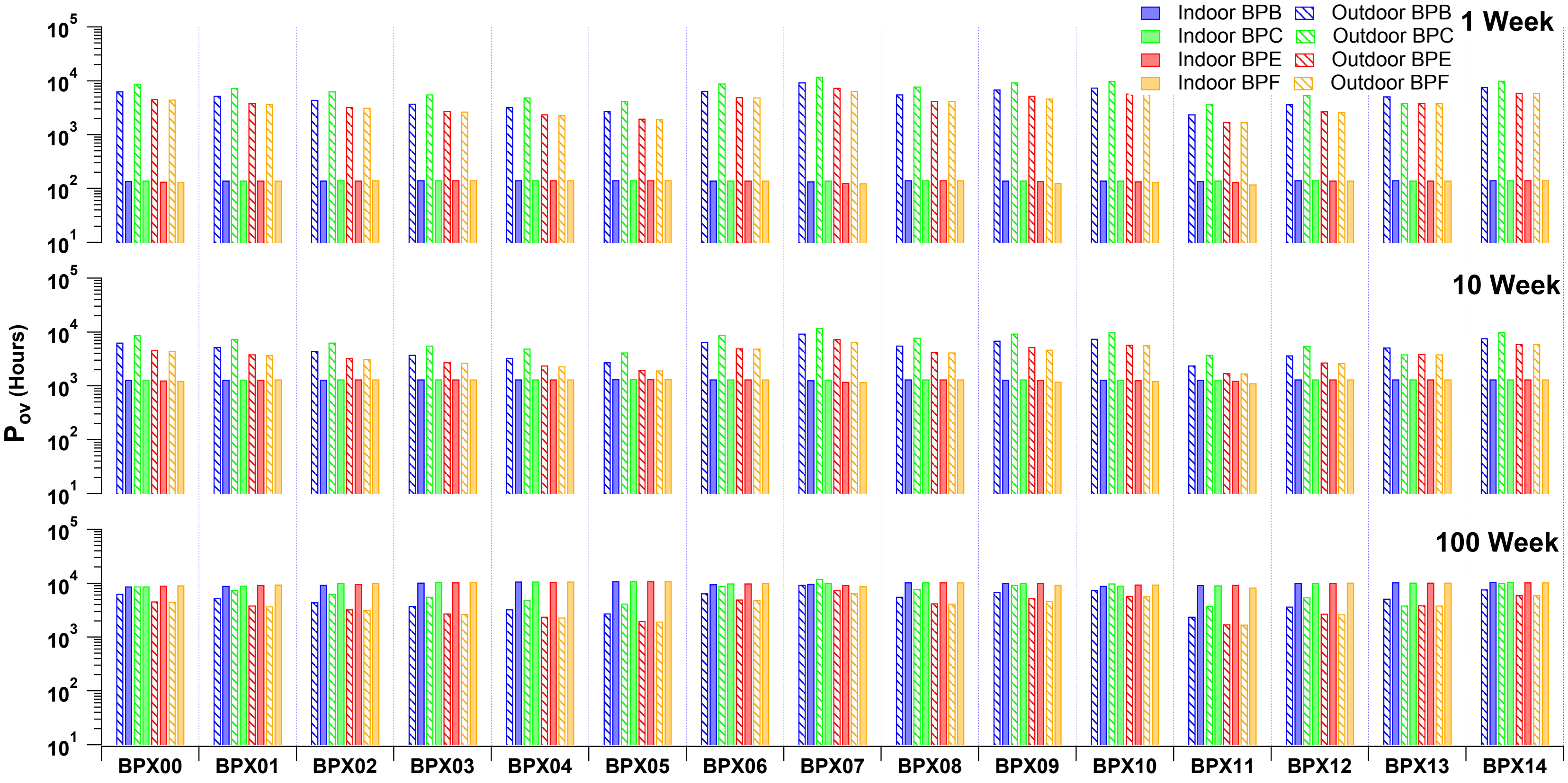
Indoor BPB
Indoor BPC
Indoor BPE
Indoor BPF
Outdoor BPB
Outdoor BPC
Outdoor BPE
Outdoor BPF
1 Week
10 Week
100 Week
$P_{ov}$ (Hours)
$10^1$
$10^2$
$10^3$
$10^4$
$10^5$
BPX00
BPX01
BPX02
BPX03
BPX04
BPX05
BPX06
BPX07
BPX08
BPX09
BPX10
BPX11
BPX12
BPX13
BPX14

242

# References


(1) Miramontes Gonzalez, P.; Li, L. Evaluating the Environmental Persistence of Liquid Crystal Monomers Indoors and Outdoors. *Environ Sci Technol Lett* **2023**, *11* (3), 216–222. https://doi.org/10.1021/acs.estlett.3c00831.

(2) Li, L.; Zhang, Z.; Men, Y.; Baskaran, S.; Sangion, A.; Wang, S.; Arnot, J. A.; Wania, F. Retrieval, Selection, and Evaluation of Chemical Property Data for Assessments of Chemical Emissions, Fate, Hazard, Exposure, and Risks. *ACS Environmental Au*. American Chemical Society September 21, 2022, pp 376–395. https://doi.org/10.1021/acsenvironau.2c00010.

(3) Estimation Programs Interface Suite™ for Microsoft® Windows. United States Environmental Protection Agency: Washington, DC, USA 2012.

(4) Mansouri, K.; Grulke, C. M.; Judson, R. S.; Williams, A. J. OPERA Models for Predicting Physicochemical Properties and Environmental Fate Endpoints. *J Cheminform* **2018**, *10* (1), 10. https://doi.org/10.1186/s13321-018-0263-1.

(5) Brown, T. N. QSPRs for Predicting Equilibrium Partitioning in Solvent–Air Systems from the Chemical Structures of Solutes and Solvents. *J Solution Chem* **2022**, *51* (9), 1101–1132. https://doi.org/10.1007/s10953-022-01162-2.

(6) Li, L.; Sangion, A.; Wania, F.; Armitage, J. M.; Toose, L.; Hughes, L.; Arnot, J. A. Development and Evaluation of a Holistic and Mechanistic Modeling Framework for Chemical Emissions, Fate, Exposure, and Risk. *Environ Health Perspect* **2021**, *129* (12), 127006. https://doi.org/10.1289/EHP9372.

(7) Fahy, W. D.; Wania, F.; Abbatt, J. P. D. When Does Multiphase Chemistry Influence Indoor Chemical Fate? *Environ Sci Technol* **2024**, *58* (9), 4257–4267. https://doi.org/10.1021/acs.est.3c08751.

(8) Morrison, G.; Lakey, P. S. J.; Abbatt, J.; Shiraiwa, M. Indoor Boundary Layer Chemistry Modeling. *Indoor Air* **2019**, *29* (6), 956–967. https://doi.org/10.1111/ina.12601.

(9) Alwarda, R.; Zhou, S.; Abbatt, J. P. D. Heterogeneous Oxidation of Indoor Surfaces by Gas-Phase Hydroxyl Radicals. *Indoor Air* **2018**, *28* (5), 655–664. https://doi.org/10.1111/ina.12476.

(10) Toby, S.; Ullrich, E. Reaction of Carbon Monoxide with Ozone: Kinetics and Chemiluminescence. *Int J Chem Kinet* **1980**, *12* (8), 535–546. https://doi.org/10.1002/KIN.550120804.

(11) Burkholder, J. B.; Sander, S. P.; Abbatt, J. P. D.; Barker, J. R.; Cappa, C.; Crounse, J. D.; Dibble, T. S.; Huie, R. E.; Kolb, C. E.; Kurylo, M. J.; Orkin, V. L.; Percival, C. J.; Wilmouth, D. M.; Wine, P. H. Chemical Kinetics and Photochemical Data for Use in Atmospheric Studies Evaluation Number 19 NASA Panel for Data Evaluation. **2020**.

(12) Yu, J.; Gong, Y.; Nair, P.; Liggio, J.; Peng, H.; Abbatt, J. P. D. Multiphase Ozonolysis of Bisphenol A: Chemical Transformations on Surfaces in the Environment. *Environ Sci Technol* **2024**, *58* (8), 3931–3941. https://doi.org/10.1021/acs.est.3c08932.

(13) Wang, J.; Zheng, M.; Deng, Y.; Liu, M.; Chen, Y.; Gao, N.; Du, E.; Chu, W.; Guo, H. Generality and Diversity on the Kinetics, Toxicity and DFT Studies of Sulfate Radical-Induced Transformation of BPA and Its Analogues. *Water Res* **2022**, *219*, 118506. https://doi.org/10.1016/j.watres.2022.118506.

(14) Hems, R. F.; Abbatt, J. P. D. Aqueous Phase Photo-Oxidation of Brown Carbon Nitrophenols: Reaction Kinetics, Mechanism, and Evolution of Light Absorption. *ACS Earth Space Chem* **2018**, *2* (3), 225–234. https://doi.org/10.1021/acsearthspacechem.7b00123.

(15) Porcar-Santos, O.; Cruz-Alcalde, A.; Bayarri, B.; Sans, C. Reactions of Bisphenol F and Bisphenol S with Ozone and Hydroxyl Radical: Kinetics and Mechanisms. *Science of The Total Environment* **2022**, *846*, 157173. https://doi.org/10.1016/j.scitotenv.2022.157173.
